# Monochromatic Blue Light Enhances Antioxidant Status and Remodels the Gut Microbiome in Association with Increased Plasma Melatonin in Broiler Chickens

**DOI:** 10.3390/antiox15091204

**Published:** 2026-09-20

**Authors:** Wanqi Tang, Zixu Wang, Yulan Dong, Jing Cao, Yaoxing Chen

**Affiliations:** State Key Laboratory of Veterinary Public Health and Safety, College of Veterinary Medicine, China Agricultural University, Haidian, Beijing 100193, China

**Keywords:** gut microbiome, monochromatic light, melatonin, oxidative stress, broiler

## Abstract

The intestinal microbiome of broiler chickens has potential to regulate host health and growth performance. Although previous studies have revealed that the intestinal microbiota composition is affected by different factors including monochromatic light, the underlying mechanisms remain poorly understood, particularly regarding the causal role of light-sensitive hormones such as melatonin. To address this gap, a 2 × 4 factorial design was adopted in the present study, with two surgical treatments (sham-operation or pinealectomy) and four light conditions (white, blue, green, and red light), to investigate whether blue light modulates gut microbiota and antioxidant status through melatonin-dependent pathways. Broilers were reared under different monochromatic light conditions. On day 3 post-hatching, we ablated circulating melatonin production by conducting a pinealectomy or control sham-operation model. Accordingly, the broilers were assigned to eight groups: white light + sham-operation (WL), white light + pinealectomy (WP), blue light + sham-operation (BL), blue light + pinealectomy (BP), green light + sham-operation (GL), green light + pinealectomy (GP), red light + sham-operation (RL), and red light + pinealectomy (RP). On day 35, blue light was found to most effectively elevate plasma melatonin, which activated the Mel 1a/Nrf2/NQO1 pathway to reduce oxidative stress and remodel the jejunal microbiota. Metagenomic analysis identified *Akkermansia muciniphila*, *Bifidobacterium longum* and *Ligilactobacillus aviarius* as key bacteria enriched in blue light. Consequently, classes of microbiota-derived metabolites like stearidonic acid and indole propionic acid triggered the variation of tryptophan, bile acid and lipid metabolism, which contributed to broiler growth promotion. Moreover, pinealectomy accompanied by plasma melatonin deprivation significantly nullified the blue-light-induced effects. These insights confirm blue light is more effective in microbiota modulation by inducing melatonin secretion and providing a new strategy for light management in the broiler industry.

## 1. Introduction

Commercial broilers have a lower feed conversion ratio and shorter lifespans compared to swine and ruminants, resulting in more efficient birds that need less food and time to reach the demand for poultry meat supply [1]. Hence, poultry health seems paramount to ensure the appropriate functioning of the body to adapt to the rapid metabolic rate and the accelerated body weight increase in fast-growing broilers. An adequate development of the gastrointestinal tract enables high utilization of nutrients and better broiler health that is essential to the success of this industry [2].

The intestinal tract harbors a diverse population of microorganisms, which are both powerful and fragile. Extensive studies revealed that the microbiota of the broiler chicken intestinal tract performs various biological functions regarding host health and productivity. Moreover, the microbiota is involved in colonization resistance to enteric pathogens through the process of competitive exclusion and the production of bacteriostatic and bactericidal substances. On the other hand, the taxonomic composition of the microbiota is susceptible to external factors such as diet intake and environment [3,4,5]. Given the spectral sensitivity of chickens, a light regime is the major determinant influencing broiler production among those environmental factors.

Broilers have highly specialized visual systems that possess light-sensitive organs including a retina and pineal gland. It is reported that broilers have six photoreceptor cells and a lipid droplet at the top of the inner segment of the cone cell that improves color discrimination in the retina [6,7,8]. Furthermore, broilers have several unique blue-light-sensitive photoreceptor proteins distributed in the pineal gland [9]. However, recent work has been conducted to understand the specific mechanism of monochromatic light on broiler growth performance focused on the pineal gland since it is an endocrine organ located under the light-transmissible cranium, whose indole metabolism is more directly influenced by light wavelength [10,11,12].

Melatonin, a vital hormone synthesized by the pineal gland, is generally considered to be a growth-regulating agent in birds. Previous work amply demonstrated developmental changes in the expression of genes involved in melatonin synthesis in the pineal gland and found green light increased the level of *cAanat* expression (a prominent gene in melatonin biosynthesis) in young broilers (14–21 days) [13,14]. Additionally, chickens (35–42 days) exposed to blue light showed a significant higher plasma melatonin level [15]. Circulating melatonin production manipulated by monochromatic light could affect multiple systems through their immune-modulating, antioxidant and anti-inflammatory functions. Indeed, numerous studies have suggested that green light enhances broiler growth at an early age, whereas blue light enhances growth in the latter stage [16,17,18].

Although light exposure has been documented to alter the gut microbiota in avian species [19,20], the specific mechanisms by which different light wavelengths influence gut microbiota in broilers remain largely unexplored. To date, however, no study has directly tested whether these light-induced changes are mediated by melatonin, nor has any study identified the downstream signaling pathway through which melatonin communicates with the gut microbiota. To address these gaps, the present study was designed to provide the first experimental evidence for the role of melatonin as a crucial mediator linking monochromatic light to broiler jejunal microbiota. We hypothesized that blue light elevates plasma melatonin, which activates the Mel1a/Nrf2/NQO1 antioxidant pathway in the jejunum, thereby reducing oxidative stress, remodeling the gut microbiome, and ultimately promoting host growth. Overall, our findings establish a mechanistic framework for understanding light–host–microbiota interactions and offer a scientific basis for optimizing light regimes to improve production efficiency in the poultry industry.

## 2. Materials and Methods

### 2.1. Study Design

This study consisted of two complementary parts: an in vivo experiment to examine the effects of light color and pinealectomy on melatonin receptor expression in the jejunum, and an in vitro experiment to dissect the downstream signaling pathway of melatonin receptor activation using the chicken small-intestinal epithelial cells (CSIECs). The in vivo experiment was conducted first, followed by the in vitro mechanistic validation. Detailed experimental procedures are described in the subsections below.

### 2.2. Animals and Experimental Design

A total of 192 post-hatching day 0 (P0) male Cobb500 chicken (Beijing Poultry Breeding Co., Ltd., Beijing, China) were randomly assigned into 4 groups and raised under 4 light-emitting diode (LED) light treatments: white light (WL, combination of wavelength, 6000K), blue light (BL, 480 nm), green light (GL, 525 nm) and red light (RL, 660 nm). All broilers were reared in a single climate-controlled room. A total of 48 pens (*n* = 4 broilers) were used, with each pen measuring 0.6 m × 0.7 m and providing a stocking density of 10 chicks/m^2^. Pens were arranged in four vertical columns, with each column consisting of four pens (one per light treatment: white, blue, green, and red). The order of light colors within each vertical column was randomized independently, such that the spatial position of each light treatment differed between columns to minimize any potential confounding by vertical location. Within each light treatment, pens were randomly assigned to either sham-operation or pinealectomy groups, ensuring that surgical status was evenly distributed across cage positions. Vertical columns were separated by 2-meter-high opaque wooden partitions to prevent light interference between adjacent columns. Each pen was equipped with an independent LED strip of the corresponding wavelength, connected to a dedicated dimmer to ensure uniform light intensity across all pens. LED bulbs providing a specific wavelength were installed at the bird head level with an illuminance of 0.025 W/m2 (FZ-A Irradiance meter, Beijing Normal University Optoelectronic Technology Co., Ltd., Beijing, China) and light-intensity calibration (ST85 Illuminance Meter, Beijing Normal University Optoelectronic Technology Co., Ltd., Beijing, China) twice a week. Although all four light colors were adjusted to the same irradiance (0.025 W/m^2^), the corresponding illuminance values differed due to wavelength-dependent spectral sensitivity: white light at 6.6 lux, blue light at 1.2 lux, green light at 15.0 lux, and red light at 6.3 lux. Each lighting regime was reset to 23L:1D from P0 to P35.

Furthermore, in half of these broilers (*n* = 96, 24 broilers per light color), pinealectomy surgery (Pinx) was conducted under intraperitoneal injection anesthesia (pentobarbital sodium 20 mg/kg body weight) on post-hatching day 3 (P3). The technique has been described previously [21]: broilers were placed on a stereotaxic apparatus, and a midline incision was created with a scalpel at the junction of the frontal and parietal bones, overlying the confluence of the sinuses. A distally based osteochondral flap was carefully elevated and reflected to expose the pineal gland, which lies between the two cerebral hemispheres and the cerebellum. The pineal gland, together with its stalk, was gently dissected and completely removed using microsurgical forceps. The osteochondral flap was then repositioned to its original site, and the skin incision was closed with interrupted sutures. All surgical procedures were performed under aseptic conditions using sterilized instruments. On the other half of the broilers, sham operation (Sham) was performed concurrently. Sham operation was identical to pinealectomy, except that the pineal gland and its stalk were only exposed instead of being removed. Postoperatively, all birds were placed in a warm and dark environment and monitored until full recovery from anesthesia (approximately 2–4 h). Food and water were withheld during this period. After fully recovery, sham-operated and pinealectomized broilers were then returned to their designated light environments and housed separately for the duration of the experiment. Mortality and any signs of distress or infection were recorded daily. No significant postoperative complications or mortality directly attributable to the surgical procedure were observed across the experimental groups.

All broilers were maintained under standard environmental conditions of temperature (started at 32 ± 1 °C in the first week, and decreased 0.5 °C daily to reach 24 °C) and relative humidity (55 ± 10%). Broilers had free access to food and water, and the diets were formulated according to National Research Council (1994) recommendations for poultry. Bird performance was evaluated by the productive performance index (PPI), calculated by the following formula, as documented [22]: PPI = ((BWG × livability)/(FCR × days of age)) × 100.

### 2.3. Sample Collection

All broilers were euthanized and sampled during the photophase (8:00–10:00 a.m.) on post-hatching day 35. Blood was first collected from the wing vein under dim light. Then, the birds were euthanized by cervical dislocation and exsanguinated to minimize the interference from blood cells in subsequent experiments. For each sampling session, eight broilers from different light color groups (including the sham-operation and pinealectomy group) were processed concurrently, with random sampling order in each group. Upon dissection, the jejunum was identified and divided into two halves (8–10 cm) at the Meckel’s diverticulum. The luminal contents of one jejunal segment were quickly collected into two sterile centrifuge tubes for subsequent metagenomic and metabolomic analyses. This segment was then cut open and gently rinsed with 0.75% sterile saline. Both the rinsed tissue and the luminal content samples were immediately snap-frozen in liquid nitrogen and stored at −80 °C. The other half of the jejunum was fixed in 4% paraformaldehyde for subsequent morphological analysis. All sample collection operations were aseptically performed on a clean bench, using 75% ethanol to prevent sample contamination. Blood samples were heparinized with 1000 UI/mL of sodium heparin (Sigma-Aldrich, H4784, St. Louis, MO, USA) and kept at 4 °C for 4 h; the plasma samples were obtained by centrifugation (4 °C, 3000 rpm, and 10 min).

### 2.4. Cell Culture and Cell Viability Assay

CSIECs were purchased from the Procell system (Wuhan, China, Cat. CP-C009). CSIECs were maintained in Dulbecco’s Modified Eagle Medium with high glucose (HyClone, SH30022.01, Logan, UT, USA), existing 10% fetal bovine serum (FBS, Gibco, 10099141, St. Louis, MO, USA), and 1% penicillin-streptomycin (Solarbio, P1400, Beijing, China) in a 5% CO_2_ incubator at 37 °C. To determine the safe and effective concentrations of melatonin and the oxidative stress inducer for subsequent mechanistic studies, we first performed a dose–response experiment. CSIECs were plated onto 96-well plates at 1 × 10^4^ cells/well and treated with various concentrations of H_2_O_2_ (0–400 µM) for 24 h. Based on the CCK-8 results, 200 µM H_2_O_2_ was selected as the optimal concentration to induce oxidative damage (inducing approximately 70% reduction in cell viability). For the formal experiments, CSIECs were pretreated with 1 µM melatonin for 30 min, followed by exposure to 200 µM H_2_O_2_ for an additional 23 h. Where indicated, cells were pre-incubated with the following inhibitors for 30 min prior to melatonin treatment: 10 µM Luzindole (a non-selective Mel1a/Mel1b antagonist, MCE, HY-101254, Monmouth Junction, NJ, USA), 1 µM 4P-P-DOT (a selective Mel1b antagonist, MCE, HY-100609, Monmouth Junction, NJ, USA), 1 µM Prazosin (a selective Mel1c antagonist, MCE, HY-B0193, Monmouth Junction, NJ, USA), or 1 µM ML385 (an Nrf2 inhibitor, MCE, HY-100523, Monmouth Junction, NJ, USA). After the treatments, 10 µL of CCK-8 solution (Yeasen, 40203ES76, Shanghai, China) was added to each well and incubated for 2 h at 37 °C in a humidified atmosphere. The absorbance was measured at 450 nm using a microplate reader (BioTek, 800TS, Winooski, VT, USA). All experiments were performed in triplicate.

### 2.5. Reactive Oxygen Species (ROS) Detection

We accessed the production of intracellular reactive oxygen species (ROS) using 2′,7′–dichlorodihydrofluorescein diacetate acetyl ester (DCFH-DA). In brief, cells were inoculated into 24-well plates at 5 × 10^4^ cells per well and respectively treated with Melatonin, H_2_O_2_ + Melatonin, H_2_O_2_ + Melatonin + Luzindole, H_2_O_2_ + Melatonin + 4P-P-DOT and H_2_O_2_ + Melatonin + Prazosin. After 18 h, the fluid was removed and incubated with DCFH-DA (Solarbio, CA1410, Beijing, China) and Hoechst 33342 (Beyotime, C1022, diluted 1:1000 with serum-free medium, Shanghai, China) for 30 min in a humidified atmosphere at 37 °C of 5% CO_2_. At last, the cells were observed and photographed by a fluorescent microscope. ROS generation was evaluated by the fluorescence signal intensity analyzed with ImageJ (version 1.54p). The fluorescence intensity was detected by a fluorescence microplate reader with excitation at 488 nm and emission at 525 nm. The relative fluorescence intensity was normalized in the control group.

### 2.6. Immunofluorescence Staining

CSIECs were cultured in 24-well plates, fixed with prepared cold 4% paraformaldehyde, blocked with 5% goat serum and incubated with primary antibody against Nrf2 (Proteintech, 16396-1-AP, 1:200 dilution, Wuhan, China) at 4 °C overnight. Subsequently, the Nrf2 antibody was detected by Alexa Fluor 594-conjugated goat anti-rabbit IgG antibody (Abcam, ab150080, 1:1000 dilution, Cambridge, UK) for 2 h. Nuclei were labeled with DAPI (Solarbio, C0065, Beijing, China). Five random fields of each well (*n* = 3 replicates/group) were captured and quantified, then the data of positive-cell staining rates were determined, and the relative density (cytoplasm/nucleus) of protein represents the nuclear translocation of Nrf2. Images were acquired by fluorescence microscopy (Olympus, BX51, Tokyo, Japan). The quantification of the integrated fluorescence intensity was performed with ImageJ software.

### 2.7. Multiplex Immunohistochemistry (mIHC)

To visualize the expression of several immune markers in the gut, multiplexed tyramide signal amplification (TSA) immunofluorescence staining was performed using a fluorescence immunohistochemistry kit from TissueGnostics (TG, TGT4C100, Vienna, Austria) on formalin-fixed and paraffin-embedded tissues. The process was conducted for multiple antibodies including IFN-γ (Abcam, ab231036, 1:200 dilution, Cambridge, UK), GZMB (Abcam, ab255598, 1:500 dilution, Cambridge, UK), and CD8 (Abcam, ab217344, 1:200 dilution, Cambridge, UK). The slides were then incubated with HRP Ms & Rb (DAKO, K406987, Glostrup, Denmark) for 20 min at room temperature before being incubated with TG TSA fluorochromes (TG520N, TG620N and TG700N, Vienna, Austria) for 10 min at room temperature. Antigen retrieval with sodium citrate buffer was conducted between cycles of tyramide signal amplification to prevent cross-reactivity. Finally, the slides were counterstained with DAPI (Solarbio, C0065, Beijing, China) for 10 min. Slide images were acquired by the Tissue FAXS.

### 2.8. PAS Staining

We visualized and assessed the number of goblet cells by periodic acid–Schiff (PAS) staining, which highlights goblet cells a purplish red. The staining procedure involved deparaffinizing and hydrating, then incubating in periodic acid solution for 10 min at room temperature, followed by rinsing with running water. The jejunal sections were next bathed with Schiff’s reagent for 15 min without light. After washing by water, the sections were counterstained with hematoxylin and analyzed. Five random slices each from 3 to 4 chickens per group were used in the analysis. The number of PAS+ goblet cells was expressed per 100 intestinal epithelial cells measured by ImageJ.

### 2.9. Enzyme-Linked Immunosorbent Assay (ELISA)

Plasma (pg/mL) and intestinal (pg/g jejunal tissue) melatonin levels were measured by a commercial ELISA kit (Chicken melatonin ELISA, SLCY, SL14404, detect range: 1–40 ng/L, Beijing, China). The experimental scheme was carried out using the manufacturer’s instructions. Each sample was tested in triplicate. The intra-assay coefficient of variation (CV) was <9%, and the inter-assay CV was <11%.

### 2.10. Antioxidant Enzyme Activity Analysis

The antioxidant activities in jejunum were detected using catalase (CAT, units/mg jejunal protein, Beyotime, S0051, Shanghai, China), superoxide dismutase (SOD, U/mg jejunal protein, Beyotime, S0101, Shanghai, China), glutathione peroxidase (GSH-Px, mU/mg jejunal protein, Beyotime, S0057, Shanghai, China), total antioxidant capacity (T-AOC, mmol/g jejunal protein, Beyotime, S0116, Shanghai, China) and malondialdehyde (MDA, μmol/mg jejunal protein, Beyotime, S0131, Shanghai, China) kits. All the tests were performed following the protocol.

### 2.11. Western Blot

Protein was extracted from cells or tissues and measured using the BCA kit (CWBio, CW0014) to qualify the protein concentration of 2 μg/μL in the sample. SDS-PAGE was used to separate the proteins. After protein (50 μg) was transferred to a PVDF membrane (Millipore, IPVH00010, Billerica, MA, USA), the membrane was blocked with 5% skimmed milk at room temperature for 1 h. Then, the membrane was incubated with a primary antibody for 2 h at room temperature. Subsequently, the membrane was incubated with horseradish peroxidase (HRP)-conjugated goat anti-rabbit IgG (CWBio, CW0103, Taizhou, China) for 2 h. Proteins were detected using Western LightningTM Plus-ECL Oxidizing Reagent Plus (Tanon, 5200, Shanghai, China). The antibodies used here are listed below: Nrf2 (110 KD, Proteintech, 16396-1-AP, Wuhan, China), Keap1 (70 KD, Proteintech, 10503-2-AP, Wuhan, China), Mel 1a (39 KD, Abcam, ab203038, Cambridge, UK), Mel 1b (36 KD, Abcam, ab203346, Cambridge, UK), Mel 1c (25 KD, Proteintech, 15767-1-AP, Wuhan, China). Protein bands were quantified using ImageJ. The protein level was normalized to the housekeeping protein β-actin (43 KD, Proteintech, 66009-1-lg, Wuhan, China).

### 2.12. Quantitative Real-Time PCR (qPCR)

Total jejunal RNA was extracted using TRIzol reagent (Vazyme, R401-01, Nanjing, China), according to the manufacturer’s instructions. For reverse transcription, 1 µg of total RNA was reverse-transcribed into complementary DNA (cDNA) using the HiScript^®^ II 1st Strand cDNA Synthesis Kit (+gDNA wiper) (Vazyme, R212, Nanjing, China) according to the manufacturer’s protocol. The genomic DNA (gDNA) removal step was performed by incubating 1 µg of total RNA with 4 µL of 4 × gDNA wiper mix at 42 °C for 2 min. Subsequently, the reverse-transcription reaction was carried out at 50 °C for 15 min, followed by enzyme inactivation at 85 °C for 2 min. The resulting cDNA was diluted 1:10 with nuclease-free water and stored at −20 °C until use. PCR amplification was performed using AceQ qPCR SYBR green master mix (Vazyme, Q111-02, Nanjing, China). Each 20 µL reaction contained 10 µL of SYBR Green master mix, 2 µL of cDNA, 0.4 µL of forward primer, 0.4 µL of reverse primer, and 7.2 µL of nuclease-free water. The cycling protocol was as follows: initial denaturation at 95 °C for 5 min, followed by 40 cycles of denaturation at 95 °C for 10 s and annealing/extension at 60 °C for 30 s. A melt curve analysis was performed after the amplification cycles to confirm the specificity of the amplification products, with the following program: 95 °C for 10 s, 60 °C for 1 min, and 95 °C for 10 s (ramping at 0.5 °C per step with continuous fluorescence acquisition). The relative expression levels of cDNA in each sample were estimated using the value of 2–ΔΔCt and normalized to the reference gene GAPDH. The reference gene GAPDH showed stable expression across all treatment groups, with Ct values consistently ranging from 18 to 19 cycles across all samples, confirming its suitability as an internal control. RT-PCR primers sequences used in the study are listed in Table 1.

### 2.13. Metagenome Analysis

Jejunal contents (*n* = 5) were collected, frozen in liquid nitrogen, and stored at −80 °C until metagenome analysis. Bacterial DNA was extracted using the commercial DNA lsolation Kit, following the manufacturer’s recommendations. For library preparation, qualified genomic DNA was fragmented by sonication. The fragmented DNA was subjected to end-repair, 3′-end adenylation, adapter ligation, and size selection. The resulting libraries were amplified and purified, and their quality was assessed using an Agilent 2100 Bioanalyzer (Agilent Technologies, Santa Clara, CA, USA). Paired-end sequencing (2 × 150 bp) was performed on an Illumina NovaSeq 6000 platform (Illumina, San Diego, CA, USA). The raw sequencing reads contain low-quality sequences. To ensure the quality of subsequent bioinformatics analysis, raw reads must be filtered to obtain clean reads for subsequent analysis. The main steps of data filtering are as follows: (1) Raw tags were filtered using fastp software (Version 0.23.4) to obtain high-quality sequencing data (Clean Tags); (2) Clean tags were aligned to the host (Gallus gallus) genome using bowtie2 (v2.5.5) to remove host contamination. The average sequencing depth per sample was represented as follows. Metagenome assembly was performed using MEGAHIT (v1.2.9), with contig sequences shorter than 300 bp filtered out. The assembly results were then evaluated using QUAST (5.0.2). Coding regions within the assembled genomes were identified by MetaGeneMark software (http://exon.gatech.edu/meta_gmhmmp.cgi, Version 3.26, accessed on 14 April 2024) with default parameters. Redundancy was removed using MMseqs2 (https://github.com/soedinglab/mmseqs2, Version 12-113e3, accessed on 14 April 2024), with a protein sequence similarity threshold set at 90% and a coverage threshold set at 80%. For statistical analysis of metagenomic data, PERMANOVA (permutational multivariate analysis of variance, 999 permutations) was performed using the adonis2 function in R (vegan package, v2.6-4) to test the effects of light color, pinealectomy, and their interaction on microbial community structure. The homogeneity of group dispersions was assessed using the betadisper function in the vegan package to confirm that significant PERMANOVA results were not driven by differences in within-group variance. Differential abundance analysis of microbial genes and pathways was performed using DESeq2 (v1.38.3), with features considered significantly different at |log2FC| > 1 and Benjamini–Hochberg adjusted *p* < 0.05. Bioinformatic analysis of the gut microbiota was carried out using the BMKCloud platform (www.biocloud.net). The GMHI was calculated according to the method originally described by Gupta et al. [23], who developed this index for evaluating health status based on species-level gut microbiome profiling in humans. The index is formulated as GMHI = log_10_ (ψ_Health-prevalent/ψ_Health-scarce), where ψ represents the collective abundance of health-associated versus disease-associated species. GMHI has been previously applied to broiler chicks, where it effectively reflected the gut microbiota health status and dysbiosis, supporting its utility in poultry studies [24]. In our study, we applied this same mathematical framework to our metagenomic dataset.

### 2.14. Untargeted Metabolomics Profiling

Metabolites from jejunal contents were extracted using the extraction solution (methanol:acetonitrile = 1:1). The LC/MS system used for metabolomics analysis consisted of a Waters Acquity UPLC I-Class PLUS system coupled with a Waters Xevo G2-XS QTof high-resolution mass spectrometer (Waters Corporation, Milford, MA, USA). The column used was purchased from Waters Acquity UPLC HSS T3 column (1.8 um, 2.1 × 100 mm) (Waters Corporation, Milford, MA, USA). The mobile phase for both positive and negative electrospray ionization (ESI) modes consisted of (A) 0.1% formic acid in water and (B) 0.1% formic acid in acetonitrile. The injection volume was 1 µL. Data were acquired in MSE mode for simultaneous collection of low- and high-energy spectra (low energy: 2 V; high-energy ramp: 10–40 V). The scan rate was 0.2 s per spectrum. ESI source parameters were: capillary voltage, ±2000 V (positive) or −1500 V (negative); cone voltage, 30 V; ion source temperature, 150 °C; desolvent gas temperature, 500 °C; backflush gas flow rate, 50 L/h; and desolventizing gas flow rate, 800 L/h. The raw data collected using MassLynx V4.2 was processed by Progenesis QI software (v2.3) for peak extraction, peak alignment and other data-processing operations, based on the Progenesis QI software online METLIN database and Biomark’s self-built library for identification, and at the same time, theoretical fragment identification and mass deviation All are within 100 ppm. After normalizing the original peak area information with the total peak area, the follow-up analysis was performed. Principal component analysis and Spearman correlation analysis were used to judge the repeatability of the samples within group and the quantity control samples. Classification and pathway information for the identified compounds were searched for in KEGG, HMDB and lipidmaps databases. According to the grouping information, to calculate and compare the difference multiples, a *t*-test was used to calculate the difference significance *p*-value of each compound. The R language package ropls was used to perform OPLS-DA modeling, and 200-times permutation tests were performed to verify the reliability of the model. The VIP value of the model was calculated using multiple cross-validation. The method of combining the difference multiple, the *p* value and the VIP value of the OPLS-DA model was adopted to screen the differential metabolites. The screening criteria are FC > 1, *p* value < 0.05 and VIP > 1. The difference metabolites of KEGG pathway enrichment significance were calculated using the hypergeometric distribution test. We performed bioinformatic analysis of metabolites on the platform BMKCloud (www.biocloud.net).

### 2.15. Statistical Analyses

All data, other than those of microbial profiling and nontargeted metabolomics, are presented as means ± standard error and were analyzed using GraphPad Prism version 9 (GraphPad Software). Experiments were performed at least in four independent biological and in at least two independent technical replicates. All data were initially tested for normality using the Kolmogorov–Smirnov test. For data involving two crossed factors, light color (white, blue, green or red) and pinealectomy (sham-operation or pinealectomy), two-way ANOVA was performed to assess the main effects of each factor and their interaction, followed by Tukey’s post hoc test for multiple comparisons. For comparisons involving only a single factor, one-way ANOVA was applied. Differences were considered statistically significant at *p* < 0.05.

## 3. Results

### 3.1. Pinealectomy Abolished the Impact of Light Wavelength on Broiler Productive Traits and Jejunal Microbiota Composition

**Figure 1:**
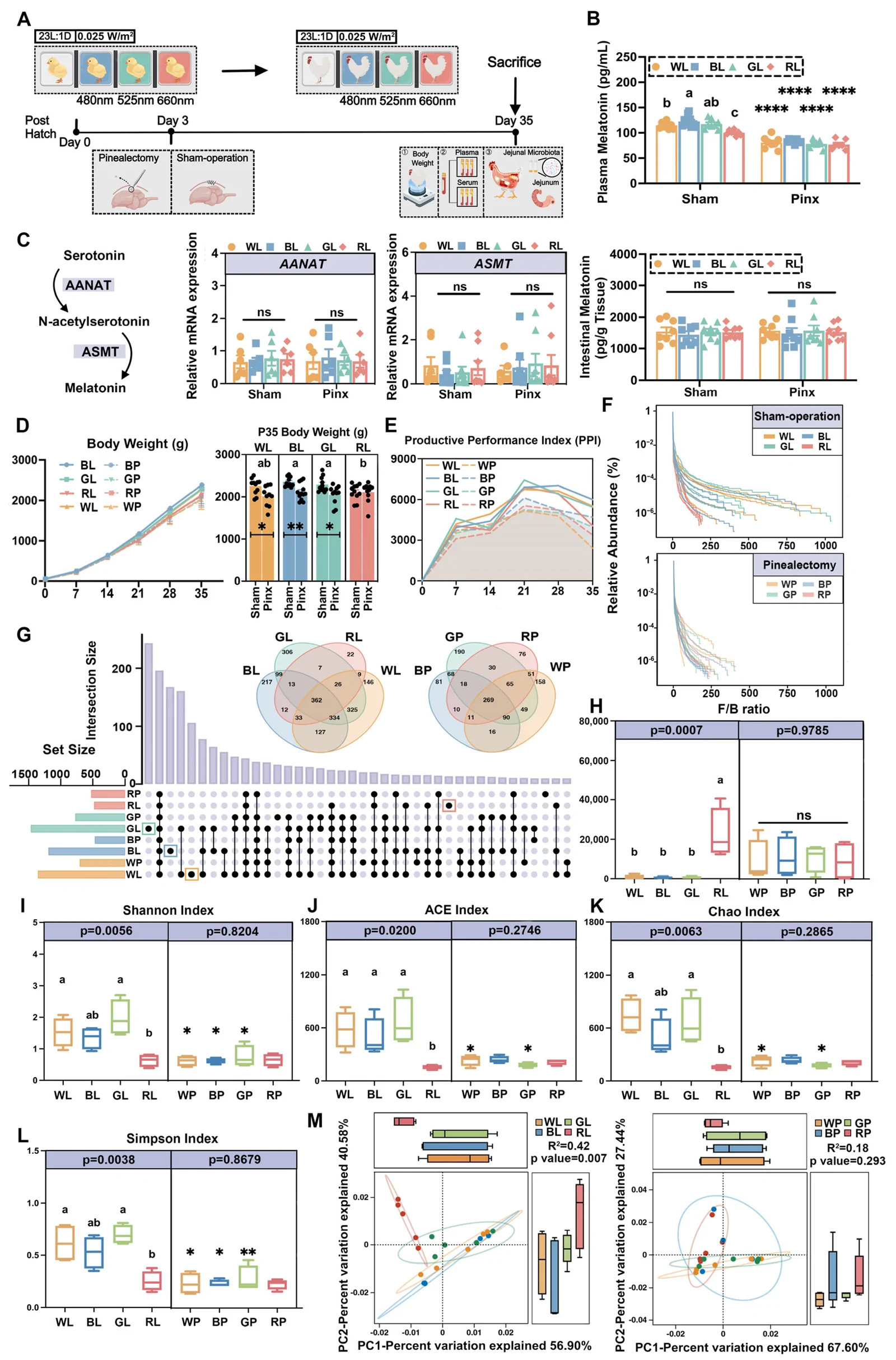
**Pinealectomy eliminates the effects of monochromatic light on broiler jejunal microbiota composition.** (**A**) Schematic and illustration of the experimental model. (**B**) Plasma and jejunal melatonin concentration in the sham-operated (Sham) and pinealectomized (Pinx) broilers under different lights. (**C**) The melatonin synthesis pathway and the mRNA expression of two rate limiting enzyme (*Aanat* and *Asmt*) during melatonin production in jejunum. (**D**) Dynamic changes in broiler body weight for 35 consecutive days and the final body weight of P35. (**E**) Productive performance index in broilers for 35 days. (**F**) Rank abundance curve (*n* = 5). (**G**) The UpSet plot represented the number of shared microbial families in Sham and Pinx groups under different monochromatic lights. (**H**) Firmicutes/Bacteroidetes ratio (F/B ratio) (*n* = 5). (**I**–**L**) Shannon, ACE, Chao and Simpson index (*n* = 5). (**M**) Principal component analysis (PCA) in Sham and Pinx groups. Data were analyzed by two-way ANOVA followed by Tukey’s post hoc test. Data are presented as the means ± SEM. Different letters on the column indicate significant differences (*p* < 0.05) between treatment groups. * represents a significant difference in the pinealectomy group (* *p* < 0.05, ** *p* < 0.01, **** *p* < 0.0001, ns = not significant) compared with the corresponding sham operations. Each data point represents one pen (*n* = 5 pens per group). Abbreviations: WL, white-light sham-operation; BL, blue-light sham-operation; GL, green-light sham-operation; RL, red-light sham-operation; WP, white-light pinealectomy; BP, blue-light pinealectomy; GP, green-light pinealectomy; RP, red-light pinealectomy.

To determine whether the effects of monochromatic light on broiler jejunal microbiota related to pineal gland-derived melatonin, we employed a chicken pinealectomy model and a sham-operation model reared under various wavelengths for 35 days (Figure 1A). Intriguingly, we observed the peak circulating melatonin level in blue-exposed chicken (14.57%, *p* = 0.0171) and the lowest melatonin in red ones (12.50%, *p* = 0.0019) compared to white-light chickens. Pinealectomized chickens showed a homogeneous pattern on plasma melatonin profiles among light color groups (*p* = 0.9342) and led to a drastic fall when compared to the sham-operated group under the same light (30.56–49.45%, *p* < 0.0001) (Figure 1B). However, pinealectomy had no significant effect on the melatonin production in gut tissue (*p* = 0.9104) (Figure 1C). Considering that broiler body weight (BW) is a typical economical trait, we next measured the BW of broilers and surprisingly investigated that higher BW alteration (9.76%, *p* = 0.0022), daily weight gain (8.28%, *p* = 0.0110) and the productive performance index (PPI) (47.46%) in blue-light broilers compared to red-light ones, which synchronized with plasma melatonin level. Moreover, pinealectomy abolished significant differences of economically trait among monochromatic lights (*p* > 0.05) and decreased the body weight (9.91%, *p* = 0.0022) and PPI (21.71%) compared to corresponding sham-operated groups (Figure 1D,E).

To testify whether monochromatic lights modulate jejunal microbiota, we characterized microbial samples from broiler chicks per wavelength to identify microbial profiles by shotgun metagenomic sequencing analyses. In sham-operated groups, blue light significantly increased the species richness and evenness compared to red lights (Figure 1F,G). Additionally, the blue-light group showed lower F/B ratio (*p* = 0.0008) (Figure 1H). Regarding alpha diversity, the ACE index was significantly higher in the blue-light group than in the red-light group (*p* = 0.0169), while the Shannon (*p* = 0.1301), Chao (*p* = 0.2236), and Simpson (*p* = 0.1730) indices showed no significant differences between the two groups (Figure 1I–L). Beta diversity, evaluated by Bray–Curtis PCoA, was clearly separated between blue- and red-light groups (*p* = 0.007, R^2^ = 0.42) (Figure 1M), indicating that gut microbiota composition changed in parallel with plasma melatonin fluctuation. To further verify whether plasma melatonin was involved in gut microbiota alternation, we next examined the microbiota composition in pinealectomized groups. As expected, alpha diversity indices among pinealectomized broilers under different light colors showed no significant differences (*p* > 0.2746). Compared with their sham-operated counterparts in the blue-light group, pinealectomized broilers showed significantly lower Shannon (*p* = 0.0338) and Simpson (*p* = 0.0352) indices, whereas the F/B ratio (*p* = 0.0872), ACE index (*p* = 0.0882), and Chao (*p* = 0.0663) index showed no significant differences (Figure 1F–L). Pinealectomized broilers also shared similar microbial communities across different light colors (*p* = 0.293, R^2^ = 0.18) (Figure 1M). Our data indicated that pineal-derived melatonin played a pivotal role in monochromatic blue light reshaping broilers’ jejunal microbiota.

### 3.2. Melatonin Mediated the Effect of Blue Light in Remodeling Microbiota Composition

**Figure 2:**
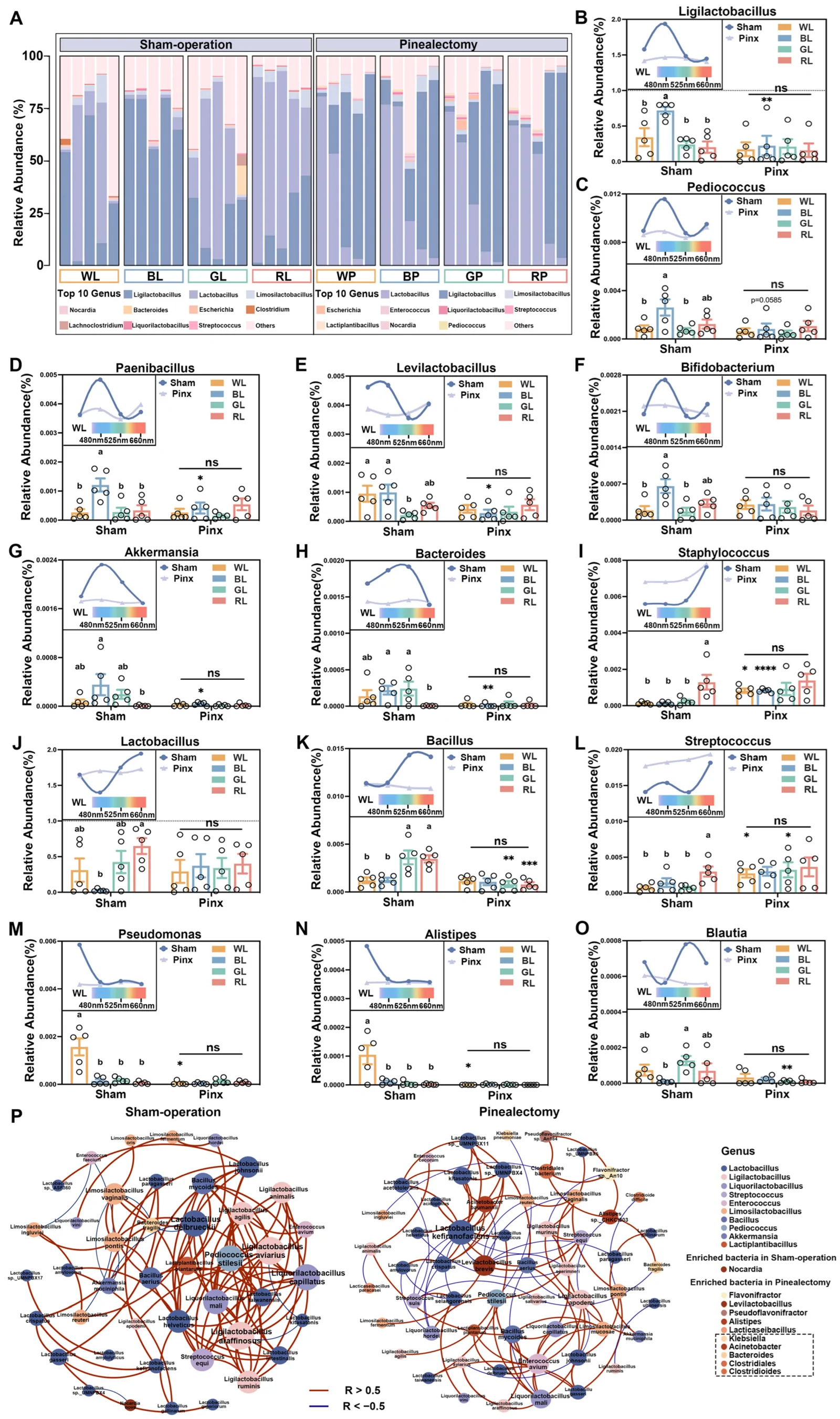
**Plasma melatonin remodels jejunal microbiota composition and modifies microbial interactions on genus level.** (**A**) Species abundance map at the genus level. One color represents one species, and the length of the color block indicates the proportion of relative abundance occupied by the species. (**B**–**O**) Relative abundance of key bacteria (*Ligilactobacillus*, *Pediococcus*, *Paenibacillus*, *Levilactobacillus*, *Bifidobacterium*, *Akkermansia*, *Bacteroides*, *Staphylococcus*, *Lactobacillus*, *Bacillus*, *Streptococcus*, *Pseudomonas*, *Alistipes*, *Blautia*) between sham-operation and pinealectomy group under monochromatic lights. (**P**) Visualization of ecological network among top 50 differential bacteria in sham-operated and pinealectomized chicken. Correlations are measured by the SparCC method and only the interaction strengths > 0.5 or <−0.5 are shown here. Dashed line represents 5 opportunistic pathogenic bacteria enriched in pinealectomy group. Data are presented as the means ± SEM. Different letters on the column indicate significant differences (*p* < 0.05) between treatment groups. * represents a significant difference in the pinealectomy group (* *p* < 0.05, ** *p* < 0.01, *** *p* < 0.001, **** *p* < 0.0001, ns = not significant) compared with the corresponding sham operations. Each data point represents one pen (*n* = 5 pens per group) Abbreviations: WL, white-light sham-operation; BL, blue-light sham-operation; GL, green-light sham-operation; RL, red-light sham-operation; WP, white-light pinealectomy; BP, blue-light pinealectomy; GP, green-light pinealectomy; RP, red-light pinealectomy.

To amplify the key bacteria enriched among different light colors, we performed a systematic review of the jejunal microbiota composition at two taxonomic levels (genus and species). At the genus level, significant variations in several taxa were observed in monochromatic lights compared to white light (Figure 2A). For the top 30 microbiota on genus level, a total 21 of them were sensitive to different wavelengths of light (*p* < 0.0422) (Figure S1). To focus on melatonin and exclude other factors caused by photostimulation, we aimed to observe the possible effects of melatonin deprivation on jejunal microbiota at the genus level. Interestingly, 14 of 21 genus (*Ligilactobacillus*, *Lactobacillus*, *Pediococcus*, *Bifidobacterium*, *Levilactobacillus*, *Paenibacillus*, *Akkermansia*, *Alistipes*, *Bacillus*, *Blautia*, *Bacteroides*, *Staphylococcus*, *Streptococcus*, *Pseudomonas*) still changed under differential wavelength light exposure and their sensitivity to specific light were immediately abolished after pinealectomy (*p* > 0.2268). Among them, *Ligilactobacillus*, *Pediococcus*, *Paenibacillus*, *Bifidobacterium*, *Akkermansia*, *Bacteroides*, *Levilactobacillus* were enriched by blue light (Figure 2B–H), together with depletion of *Streptococcus*, *Lactobacillus*, *Bacillus* and opportunistic pathogen *Staphylococcus* (which were enriched in red light) (Figure 2I–L). In addition, broilers in white-light group displayed an elevated trend of *Pseudomonas* (another opportunistic pathogen) and *Alistipes* (Figure 2M,N), while *Blautia* was more prevalent under green light (Figure 2O). To identify the distinctive features of microbiota composition and their interactions after systemic melatonin withdrawal, we constructed ecological networks which suggested that both cooperative interactions and antagonistic interactions such as competition predominate in jejunal microbial communities were strikingly different. Communication between bacterial species was weaker and progressively inclined towards microbiota dysbiosis in pinealectomized flocks. Particularly, we observed that some co-excluding interactions occurred only when five opportunistic pathogenic bacteria enriched (*Klebsiella*, *Acinetobacter*, *Bacteroides*, *Clostridiales*, *Clostridioides*) in pinealectomy group (Figure 2P). Our data revealed that alteration of microbial interactions was tightly associated with plasma melatonin.

**Figure 3:**
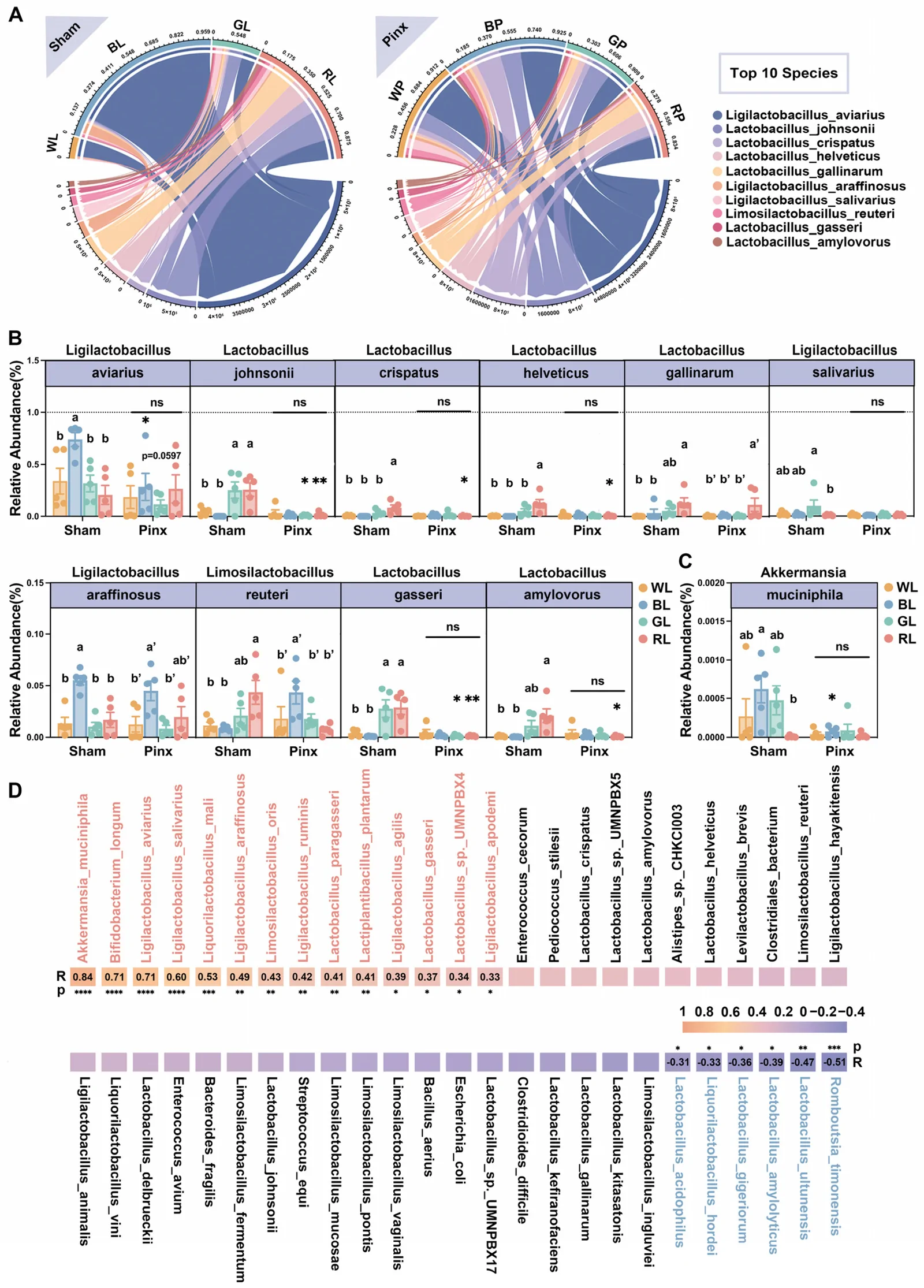
**Blue light regulates melatonin-sensitive microbiota composition to promote jejunal microbial homeostasis.** (**A**) Chord diagram of the jejunal microbiota composition at the species level in sham-operation and pinealectomy groups. (**B**,**C**) Relative abundance of top 10 species (*Ligilactobacillus aviarius*, *Lactobacillus johnsonii*, *Lactobacillus crispatus*, *Lactobacillus helveticus*, *Lactobacillus gallinarum*, *Ligilactobacillus salivarius*, *Ligilactobacillus araffinosus*, *Limosilactobacillus reuteri*, *Lactobacillus gasseri*, *Lactobacillus amylovorus*) and *Akkermansia muciniphila* between sham-operation and pinealectomy groups under monochromatic lights. (**D**) Spearman’s correlation analysis between plasma melatonin concentration and top 50 differential bacteria abundance. Data are presented as the means ± SEM. Different letters on the column indicate significant differences (*p* < 0.05) between treatment groups. * represents a significant difference in the pinealectomy group (* *p* < 0.05, ** *p* < 0.01, *** *p* < 0.001, **** *p* < 0.0001, ns = not significant) compared with the corresponding sham operations. Each data point represents one pen (*n* = 5 pens per group). Abbreviations: WL, white-light sham-operation; BL, blue-light sham-operation; GL, green-light sham-operation; RL, red-light sham-operation; WP, white-light pinealectomy; BP, blue-light pinealectomy; GP, green-light pinealectomy; RP, red-light pinealectomy.

We then assessed the top 10 core microbiota species abundances in all groups (Figure 3A). As shown in Figure 3B, *Ligilactobacillus aviarius* which accounted for the most abundant annotated species, was dominated in blue light. *Ligilactobacillus salivarius* seemed to be more abundant in green light. These differences among different light colors were noteworthily eliminated by pinealectomy. However, another member of *Ligilactobacillus* genus, *Ligilactobacillus araffinosus* remained its sensitivity to blue light after pineal gland removal. Apart from bacteria enriched in blue and green light, *Lactobacillus johnsonii*, *Lactobacillus crispatus*, *Lactobacillus helveticus*, *Lactobacillus gallinarum*, *Lactobacillus gasseri*, *Limosilactobacillus reuteri*, *Lactobacillus amylovorus* were identified to be the most frequently detected species in red light. Five species, namely *Lactobacillus johnsonii*, *Lactobacillus crispatus*, *Lactobacillus helveticus*, *Lactobacillus gasseri*, *Lactobacillus amylovorus* only remained photosensitive without pinealectomy. Moreover, *Akkermansia muciniphila* was an outstanding one out of top 10 species for its extraordinary sensitivity to blue light (Figure 3C). From species-level characterization, we inferred that melatonin could at least partially restore chicken microbiota homeostasis. In order to identify which bacterium was driven by plasma melatonin, we performed Spearman’s correlation analysis between plasma melatonin and the top 50 species-level bacteria, revealing that only 14 bacteria, especially the *Akkermansia muciniphila*, *Bifidobacterium longum* and *Ligilactobacillus aviarius*, were positively correlated with plasma melatonin and six bacteria, especially the *Romboutsia timonensis*, *Lactobacillus ultunensis* and *Lactobacillus amylolyticus*, were negatively correlated with plasma melatonin (Figure 3D). In this manner, blue-light exposure was associated with higher plasma melatonin levels and a concomitantly higher Gut Microbiome Health Index (GMHI) compared with red light (40.38% increase, *p* = 0.0180) and pinealectomized groups (57.20% increase, *p* = 0.0063) (Figure S2). The GMHI is a calculated microbiome index based on the relative abundance of beneficial versus detrimental bacterial taxa and could be a microbial composition metric. Overall, these findings established that blue light manipulated melatonin-driven microbiota composition to promote jejunal microbiota homeostasis.

### 3.3. Blue-Light Amplification of Melatonin Could Impact Jejunal Metabolism in Broilers

**Figure 4:**
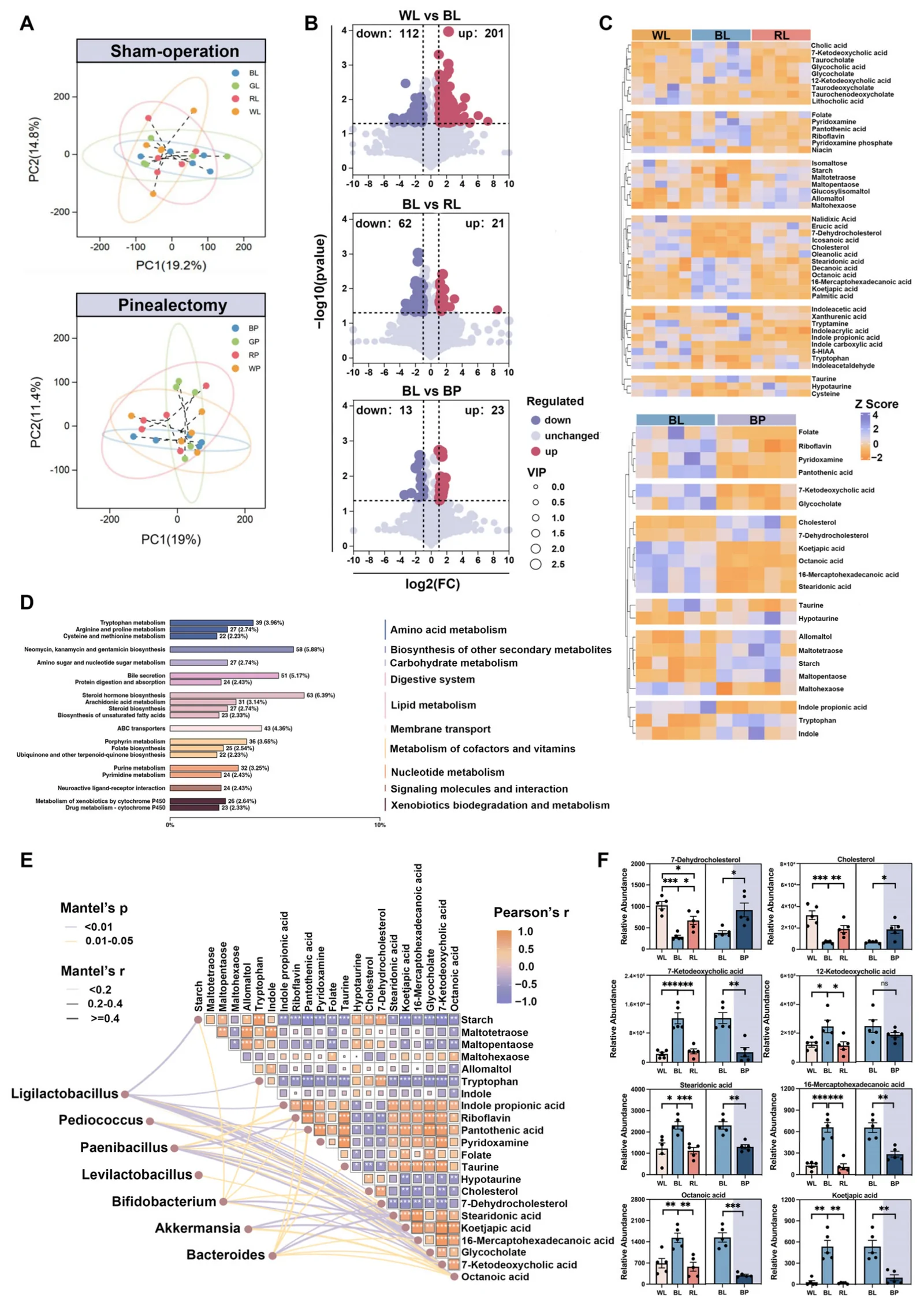
**Blue light alters jejunal microbiota-related metabolites in broilers.** (**A**) Metabolic profile between sham-operated and pinealectomized broilers analyzed by principal component analysis (PCOA). (**B**) Volcano plot of differential metabolite abundance in WL and BL, BL and RL, BL and BP groups, respectively. (**C**) Differential metabolites from nontarget metabolism between control, highest melatonin (BL) and lowest melatonin (RL) group, and sham-operation and pinealectomy group under blue light. (**D**) Enrichment analysis of differential metabolites. (**E**) Mantel test shows the association between blue-light-driven microbiota and differential metabolites. (**F**) Relative abundance of differential metabolites (7-Dehydrocholesterol, Cholesterol, 7-Ketodeoxycholic acid, 12-Ketodeoxycholic acid, Stearidonic acid, 16-Mercaptohexadecanoic acid, Octanoic acid, Koejapic acid). Data are presented as the means ± SEM. * represents a significant difference in the pinealectomy group (* *p* < 0.05, ** *p* < 0.01, *** *p* < 0.001, ns = not significant) compared with the corresponding sham operations. Each data point represents one pen (*n* = 5 pens per group). Abbreviations: WL, white-light sham-operation; BL, blue-light sham-operation; GL, green-light sham-operation; RL, red-light sham-operation; WP, white-light pinealectomy; BP, blue-light pinealectomy; GP, green-light pinealectomy; RP, red-light pinealectomy.

Given that the metabolic potential of gut microbes has been shown to regulate host metabolism in broiler chicken growth. We performed untargeted metabolomics profiling of jejunal content and principal component analyses revealed that, whether to withdraw systemic melatonin or not, the overall metabolite composition in each group showed a resemblance to each other (Figure 4A). However, we observed significant differences in the metabolite concentration especially in broilers with the highest plasma melatonin levels (*p* < 0.05, |log2FC| > 2) (Figure 4B). To identify the altered metabolites associated with plasma melatonin, we focused on blue and red light which induced maximum and minimum melatonin production. Compared to the conventional white light, 46 metabolites were found fluctuating in metabolite content of jejunum under different light spectra. To verify the specific effect of melatonin on metabolite composition, we analyzed metabolite composition after pinealectomy under blue light. Interestingly, we noticed that there were 22 metabolites overlapping with the previous 46 differential metabolites (Figure 4C). These results indicated that plasma melatonin exerted moderate, but statistically significant effect on the jejunal concentration of these metabolites. We next performed Kyoto Encyclopedia of Genes and Genomes (KEGG) signaling pathway enrichment analysis to prioritize relevant biological pathways and this enrichment analysis yielded 20 signaling pathways, mainly involved in amino acid metabolism and lipid metabolism (Figure 4D). To link these metabolites with potential metabolic activities of gut microbes, we performed Mantel-test network heat map which highlighted seven melatonin-sensitive genera, *Ligilactobacillus*, *Pediococcus*, *Paenibacillus*, *Levilactobacillus*, *Akkermansia*, *Bacteroides* and *Bifidobacterium* were found to be closely related to the lipid metabolism pathway as well as the tryptophan metabolism pathway (Figure 4E). Here, we found that plasma melatonin upregulated indole propionic acid (IPA) by 88.82% (*p* = 0.0032), a microbiota-specific metabolite from tryptophan. Notably, melatonin itself, as a product of tryptophan metabolism, did not differ significantly among groups, further indicating that pinealectomy did not affect in situ jejunal melatonin biosynthesis (*p* = 0.8265) (Figure S3). Furthermore, plasma melatonin rejuvenated jejunal microbiota, which was accounted for significant decrement in the levels of cholesterol (*p* = 0.0003) and its precursor dehydrocholesterol (*p* < 0.0001) in the jejunum of broiler chickens, and increased the levels of cholic acid derivatives such as 7-Ketodeoxycholic acid (*p* < 0.0001), Glycocholate (*p* = 0.0083), and fatty acids such as Koetjapic acid (*p* < 0.0001), 16-Mercaptohexadecanoic acid (*p* < 0.0001), Stearidonic acid (*p* = 0.0021) and Octanoic acid (*p* = 0.0015) (Figure 4F).

In summary, we have found that melatonin mediated monochromatic light affects the composition of the microbiota, the interactions between the microbiota, and the metabolites in the broiler jejunum, which in turn affects the microbial homeostasis of the broiler jejunum, enriching probiotic bacteria such as *Akkermansia muciniphila*, *Bifidobacterium longum*, *Ligilactobacillus aviarius*, *Ligilactobacillus salivarius* and other probiotics improved broiler growth.

### 3.4. Melatonin Mediated the Effect of Monochromatic Light on Gut Microbial Homeostasis by Reducing Oxidative Stress in Broilers

**Figure 5:**
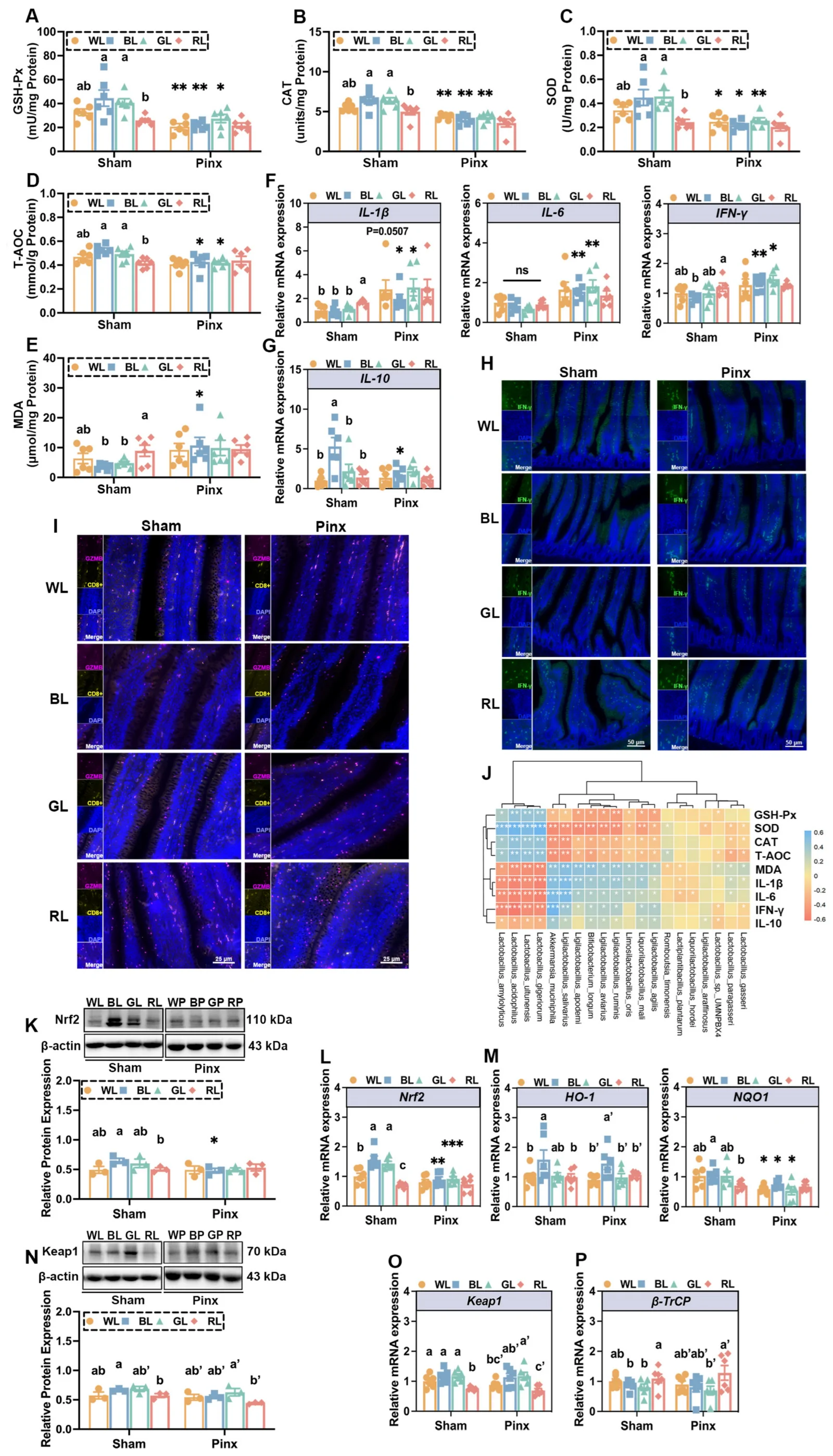
**Plasma melatonin modulates gut microbial homeostasis by reducing oxidative stress.** (**A**–**E**) GSH-Px, SOD, CAT, T-AOC and MDA levels in jejunum. (**F**,**G**) Relative mRNA expression of pro-inflammatory (*IL1β*, *IL6*, *IFNγ*) and anti-inflammatory (*IL10*) cytokines in broiler jejunum. (**H**,**I**) Multispectral image of broiler jejunum exposed to various lights (green, IFNγ; yellow, GZMB; blue, DAPI; purple, CD8). (**J**) Association analysis of melatonin-sensitive bacteria with oxidative status by Spearman correlation. (**K**) Western blots and quantification of Nrf2 expression in the broiler jejunum. (**L**,**M**) Relative mRNA expression of *Nrf2* and its target antioxidative genes (*HO-1*, *NQO1*). (**N**) Western blots and quantification of Keap1 expression in the broiler jejunum. (**O**,**P**) Relative mRNA expression of *Keap1* and *β-TrCP*. Data from 4 light groups are assessed by ANOVA followed by a Tukey multiple comparison test and the 2 groups between pinealectomy and sham-operation under one light condition were compared using unpaired Student t test. Data are presented as the means ± SEM. Different letters on the column indicate significant differences (*p* < 0.05) between treatment groups. * represents a significant difference in the pinealectomy group (* *p* < 0.05, ** *p* < 0.01, *** *p* < 0.001, ns = not significant) compared with the corresponding sham operations. Each data point represents one pen (*n* = 6 pens per group). Abbreviations: WL, white-light sham-operation; BL, blue-light sham-operation; GL, green-light sham-operation; RL, red-light sham-operation; WP, white-light pinealectomy; BP, blue-light pinealectomy; GP, green-light pinealectomy; RP, red-light pinealectomy.

To further understand whether melatonin mediated the influence of monochromatic light on microbial homeostasis in the broiler gut by reducing oxidative stress, we measured the antioxidant capacity of broiler jejunum under different light colors treated with or without pinealectomy using reagent kits targeting glutathione peroxidase (GSH-Px), catalase (CAT), superoxide dismutase (SOD), total antioxidant capacity (T-AOC) and malondialdehyde (MDA) content. Coincide with plasma melatonin fluctuations, pinealectomy decreased the activities of GSH-Px (51.34%, *p* = 0.0010) (Figure 5A), CAT (38.47%, *p* = 0.0153) (Figure 5B), SOD (51.21%, *p* = 0.0009) (Figure 5C) and T-AOC (19.05%, *p* = 0.0050) (Figure 5D) and augmented the level of MDA (66.03%, *p* = 0.0260) (Figure 5E) under blue light. Conversely, the enhancement was observed in terms of antioxidant activities among sham-operated groups especially broilers in blue light (*p* < 0.0395). Additionally, plasma melatonin deficiency increased the gene expression coding for pro-inflammatory cytokines (*IL-1β*, *IL-6*, *IFN-γ*) (Figure 5F) and reduced anti-inflammatory cytokine (*IL-10*) (Figure 5G) expression in the jejunum of broiler chickens reared under blue light (*p* < 0.0023). Coherently, multiplex immunohistochemistry showed that blue light significantly decreased the number of IFN- and CD8-positive cells and the expression of granzyme in the jejunum of broiler chickens compared with other light colors. Conversely, inflammatory cells were increased among pinealectomized chicken, but there was no significant difference in granzyme expression (Figure 5H,I). It can be hypothesized that the relationship between blue light and broiler gut microbial homeostasis may be driven by plasma melatonin concentration, which exerted its antioxidative effect to limit inflammation (Figure 5J).

Melatonin is documented to protect against oxidative stress by activating Nrf2 protein and its downstream target proteins. Here, we first examined the gene (56.10%, *p* < 0.0023) and protein (30.73%, *p* < 0.0134) expression levels of Nrf2 and found that the expression level of Nrf2 was significantly increased in the jejunum of broilers in the blue-light group. And this trend was eliminated by pinealectomy (25.76–42.40%, *p* < 0.0468). The increase in Nrf2 correspondingly up-regulated the gene expression of *NQO1* (29.64%, *p* = 0.0165), without affecting the expression of *HO-1* (*p* = 0.7310) (Figure 5K–M). However, for the upstream regulatory molecules of Nrf2, *Keap1*, *β-TrCP* expression is not regulated by melatonin (*p* > 0.7033) (Figure 5N–P). Taken together, we suggest that melatonin exerts its antioxidant effects and inhibits the expression of inflammatory factors through the activation of Nrf2/NQO1 pathway, thereby regulating its microbial homeostasis.

### 3.5. Melatonin Exerts Its Antioxidant Effects by Activating Mel1a/Nrf2/NQO1 Pathway

**Figure 6:**
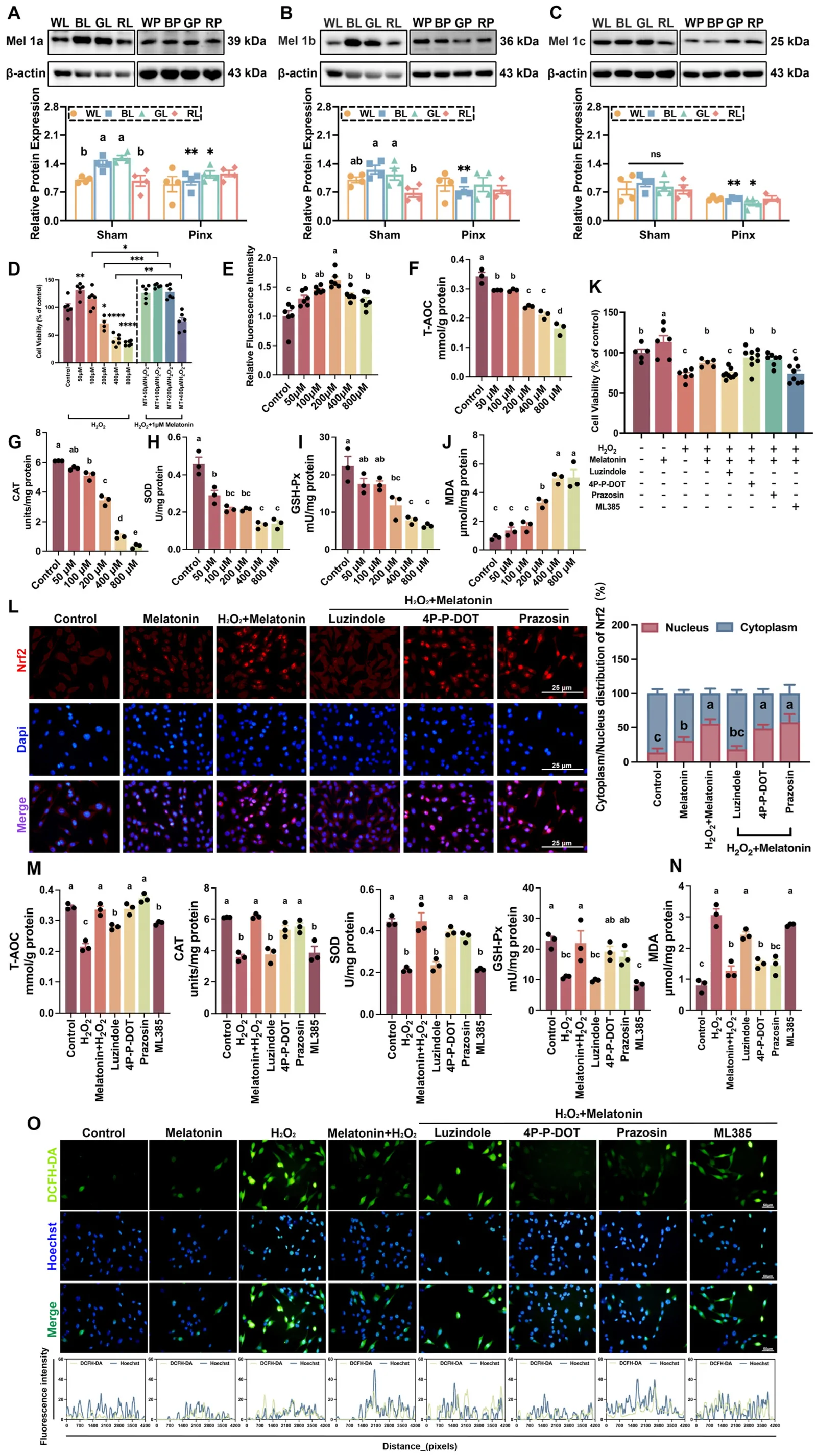
**Melatonin activates Mel1a/Nrf2/NQO1 to limit oxidative stress.** (**A**–**C**) Western blots and quantification of Mel1a, Mel1b, Mel1c expression in the broiler jejunum. (**D**) Cell viability assay of CSIEC which were treated with different concentrations of H_2_O_2_ (50, 100, 200, 400 and 800 μM). (**E**) Relative fluorescence intensity of ROS in CSIEC treated with different concentrations of H_2_O_2_ (50, 100, 200, 400 and 800 μM). (**F**–**J**) T-AOC, CAT, SOD, GSH-Px, MDA level in CSIEC treated with different concentrations of H_2_O_2_ (50, 100, 200, 400 and 800 μM). (**K**) The effective inhibition of Luzindole and ML385 on CSIEC treated with melatonin and H_2_O_2_. (**L**) Representative fluorescent images of Nrf2 and quantification of the cytoplasm/nucleus distribution (%) in CSIEC (red, Nrf2; blue, Hoechst). (**M**,**N**) T-AOC, CAT, SOD, GSH-Px, MDA level in CSIEC treated with Melatonin, H_2_O_2_, Luzindole, 4P-P-DOT, Prazosin and ML385 in CSIEC. (**O**) 2’,7’-dichlorodihydrofluorescein diacetate (DCFH-DA) was used to observe ROS generation after treatment with Melatonin, H_2_O_2_, Luzindole, 4P-P-DOT, Prazosin and ML385 in CSIEC and fluorescence intensity visualizations were showed below (green, DCFA-DA; blue, Hoechst). Data are presented as the means ± SEM. Different letters on the column indicate significant differences (*p* < 0.05) between treatment groups. * represents a significant difference in the pinealectomy group (* *p* < 0.05, ** *p* < 0.01, *** *p* < 0.001, **** *p* < 0.0001, ns = not significant) compared with the corresponding sham operations. Abbreviations: WL, white-light sham-operation; BL, blue-light sham-operation; GL, green-light sham-operation; RL, red-light sham-operation; WP, white-light pinealectomy; BP, blue-light pinealectomy; GP, green-light pinealectomy; RP, red-light pinealectomy.

It is previously reported that melatonin’s ability to versatile functions was related to its receptor-dependent actions. To gain molecular insights into the role of melatonin receptor in the regulation of the redox status of broilers’ jejunum, we next examined the expression of melatonin receptors in the jejunum of broiler chickens under different light colors. we found that only the protein expression of two membrane receptors, Mel1a (30.14%, *p* = 0.0251) and Mel1b (41.08%, *p* = 0.0073), was consistent with changes in plasma melatonin levels among light colors while Mel1c had no significant difference (*p* = 0.7479) (Figure 6A–C). This also suggested that melatonin may act through these two membrane receptors. To dissect the mechanistic pathway underlying the in vivo observation that melatonin receptor expression correlated with melatonin levels, we next performed in vitro experiments on chicken small intestinal epithelial cells (CSIEC) in vitro. Our data showed that 200 µM H_2_O_2_ can significantly upregulate cellular ROS levels while reducing its antioxidant capacity, consistent with the production of MDA (*p* < 0.0001). As expected, we found that melatonin provided cytoprotective effect on CSIEC treated with hydrogen peroxide (Figure 6D–J). Since protein expression of melatonin receptors Mel 1a and Mel 1b were verified to fluctuated in different light regimes in broilers, we tended to examine melatonin receptor-agonist to identify the specific antioxidant receptor. Intriguingly, Luzindole (a non-selective Mel 1a and Mel 1b inhibitor) applied together with melatonin significantly suppressed melatonin-dependent protective effect (15.63%, *p* = 0.0011), while 4P-P-DOT (a specific Mel 1b inhibitor) (*p* = 0.1711) and Prazosin (a specific Mel 1c inhibitor) (*p* = 0.2502) did not eliminate this influence on isolated CSIEC. Furthermore, the protective effect could be significantly interfered by ML385 (an Nrf2 blocker) correspond with the results of the in vivo assay (15.37%, *p* = 0.0170) (Figure 6K, Figure S4). Thus, we propose that melatonin may activate Nrf2 by interacting with Mel 1a, thereby promoting antioxidant defense to improve cell viability. The most canonical mechanism described for the activation of the transcription factor Nrf2 is based on its translocation from the cytosol to the nucleus. Assessed as an accumulation of Nrf2 in nucleus, Nrf2 nuclear translocation was modulated by melatonin as detected by immunofluorescence. We also found that Luzindole (67.12%, *p* = 0.0002) rather than 4P-P-DOT (*p* = 0.1592) and Prazosin (*p* = 0.7581) strongly suppressed Nrf2 nuclear translocation mediated by melatonin (Figure 6L). Melatonin promotes Nrf2 nuclear translocation to govern the gene expression of endogenous antioxidant synthesis and ROS-eliminating enzymes. Additional work was needed to understand whether melatonin could attenuate the oxidative stress. We next evaluated the antioxidant capacity of CSIEC among different treatment groups and found that melatonin significantly reduced intracellular oxidative stress levels, as evidenced by elevated T-AOC, CAT, SOD, GSH-Px, and decreased MDA (*p* < 0.0001). Moreover, this antioxidant protective effect could be markedly blocked by Luzindole and ML385 (*p* < 0.05) (Figure 6M,N). As measured by DCFH-DA assay on CSIEC, we observed that melatonin treatment reduced oxidative fluorescence, indicating a decrease in intracellular ROS levels. Simultaneously, Luzindole and ML385 respectively inhibited melatonin-dependent ROS reduction (Figure 6O). These findings suggest that melatonin is associated with reduced intracellular oxidative status, potentially through activation of Nrf2 and its downstream antioxidative genes.

## 4. Discussion

Lighting is of foremost importance in poultry operations. Previous studies have indicated that poultry color perception of monochromatic lights mediates bird physiological function and growth performance [25,26,27]. Gut microbiota is fundamental for maintaining gut health and influences the overall performance in broilers. Here, we demonstrate that monochromatic light shapes the gut microbiota by modulating systemic melatonin. Melatonin biosynthesis in chickens is primarily regulated by the pineal gland since it contains photoreceptive molecules and adrenergic receptors. Through perceiving and transmitting environmental light stimuli by putative blue-light photoreceptors (pinopsin and Cry4), the pineal gland is capable of generating circadian oscillators to regulate the rhythmic production of melatonin [9,28,29]. Additionally, norepinephrine (NE) released from sympathetic nerve fibers upon photostimulation activates alpha-2 adrenergic receptors to inhibit melatonin biosynthesis by regulating arylalkylamine-N-acetyltransferase (AANAT, an enzyme responsible for melatonin production) activity [30,31,32,33].

In our study, we found that broiler jejunum could also produce melatonin. Generally, pineal-derived melatonin circulates systemically and acts on the intestinal microvascular endothelial cells, lamina propria lymphocytes and the basement membrane of intestinal epithelial cells facing the vascular side. In contrast, intestinal melatonin is synthesized in situ and acts locally to safeguard the lumen interface [34]. Our finding provided a correlational framework, which effectively dissociated the influence of locally produced intestinal melatonin from that of systemically pineal-derived melatonin in our model. This dissociation allowed us to rigorously test our central hypothesis [35,36]. We proposed that blue light improves the gut microbiota composition by inducing a peak plasma melatonin level. A key prediction of this hypothesis is that pinealectomy should abolish the beneficial effect of blue light on the microbiota. Our results were entirely consistent with this prediction. The persistence of gut microbiota dysbiosis following pinealectomy, along with the stability level of gut tissue melatonin, strongly supported that the “light wavelength–pineal–plasma melatonin” axis is specific, without interfering with the intestine’s own synthesis. Taken together, our data suggest that monochromatic blue light may influence the gut microbiome at least in part through a central neuroendocrine pathway: it induced pineal-derived melatonin secretion and circulation to the jejunum. Then, plasma melatonin bound to melatonin receptors, which widely expressed on intestinal mucosal cells, thereby regulating jejunal redox states and ultimately reshaping the microbial community.

Melatonin is an astonishingly versatile protector against oxidative stress. Indeed, melatonin can activate Nrf2 by preventing the phosphorylation and nuclear export of Nrf2. This action effectively stabilizes and increases the Nrf2 nuclear retention, which is well known to render cellular resistance against oxidative stress and enhance transcription of target antioxidant genes in the gut. Excessive Nrf2 needs to be triggered to its degradation, which is classified into two main ways (Keap1 and β-TrCP) [37]. In this experiment, we observed that melatonin promotes Nrf2 translocation to upregulate target antioxidant genes expression (NQO1) instead of inhibiting the degradation of Nrf2. Based on these observations, we propose that the antioxidative effect of melatonin in our model is at least partially mediated via Nrf2 activation. However, as we did not perform Nrf2 knockdown or inhibition experiments in vivo, the quantitative contribution of this pathway to the overall antioxidant phenotype remains to be determined.

In fact, reduced oxidative stress enables intestinal colonization by certain bacteria. Gut microbiota composition is under the influence of elevated oxidative stress, which induces aerobic bacteria increasing and anaerobic bacteria decreasing [38,39]. Previous work suggests to stratify these gut microbes into two main types according to their response to oxidative stress. One is oxidative stress-sensitive (Ox-S) bacteria, which have susceptibility to oxidative stress. The other is called oxidative stress-resistant (Ox-R) bacteria, which exhibit strong oxidative stress resistance [40]. Here, we have confirmed plasma melatonin exhibits antioxidants activity through enzymatic mechanisms under Nrf2 activation. These enzymes scavenge ROS to lower gut redox potential, increase Ox-R bacteria (*Bifidobacterium* and *Ligilactobacillus*), while decreasing facultatively anaerobic bacteria (*Lactobacillus*) that are sensitive to ROS [41,42,43]. In addition, ROS causes lipid peroxidation, which leads to generation of some toxic chemical reactive aldehydes including malondialdehyde (MDA) [44]. MDA, as a reactive end product of lipid peroxidation, has a high tendency to increase *Escherichia coli* colonization to induce and exacerbate intestinal inflammation [45]. Except for Ox-R bacteria, taking control of excessive oxidative stress is also associated with immune modulation through maintenance of Ox-S bacteria (*Akkermansia muciniphila*) [46,47]. These studies confirmed oxidative stress could manipulate the microbiota composition through affecting Ox-S and Ox-R bacteria, prompting us to further speculate specific bacteria species related to plasma melatonin alternation. Extending this rationale to our findings, we propose that the selective enrichment of *Akkermansia muciniphila*, *Bifidobacterium longum*, and *Ligilactobacillus aviarius* under blue light can be explained by their distinct physiological traits and ecological interactions. First, although *Akkermansia muciniphila* is classified as Ox-S, its reliance on mucin as a primary carbon source may render it particularly responsive to melatonin-induced improvements in intestinal barrier integrity and mucus turnover. The reduced oxidative stress may preserve the mucus layer, providing a substrate-rich niche for this mucin-degrading bacterium. Second, *Bifidobacterium longum* and *Ligilactobacillus aviarius* are Ox-R bacteria that possess robust antioxidant defense systems, including endogenous superoxide dismutase (SOD) and catalase, enabling them to thrive under the moderately reduced redox environment created by melatonin [48,49]. This interpretation is further supported by recent evidence demonstrating that melatonin elicits species-specific transcriptional and phenotypic responses in *Bifidobacteria*, with *Bifidobacterium bifidum* showing the most pronounced molecular interaction with melatonin [50]. Notably, melatonin has been shown to upregulate genes involved in carbohydrate metabolism, membrane biogenesis, and pilus-mediated adhesion—traits that are crucial for gut colonization and host interaction. In addition, melatonin significantly enhances the adhesion of *Bifidobacterial* cells to intestinal epithelial cells, suggesting that melatonin not only modulates redox status but may also directly influence the colonization fitness of specific beneficial taxa. Third, ecological synergy among these taxa cannot be overlooked: mucin degradation by *Akkermansia* may liberate host-derived carbohydrates that serve as growth substrates for *Bifidobacterium* and *Ligilactobacillus*, forming a cooperative network that collectively prospers under blue-light conditions [51]. We acknowledge that these mechanistic interpretations require further validation through in vitro co-culture and gnotobiotic experiments. Nevertheless, our data suggest that the interplay between melatonin-mediated redox modulation and taxon-specific physiological traits likely underpins the observed enrichment pattern.

In our study, this speculation was substantiated by the distinct microbial community under different light wavelength. Specifically, we focused on the *Firmicutes*/*Bacteroidetes* (F/B) ratio, a key indicator closely associated with gut microbiome homeostasis and growth performance in fast-growing broilers. We observed a moderately lower F/B ratio in blue-light broilers than that of red-light broilers. This result may reflect a microbial community with higher microbiome diversity that favors gut homeostasis and optimizes growth performance. Our F/B ratio of blue-light treatment was accompanied by an increase in beneficial bacteria genera of *Ligilactobacillus*, which suggested that the microbial ecosystem reorganizes towards a state that likely supports more efficient nutrient metabolism and enhanced host antioxidant capacity, thereby contributing to the improved growth performance as we observed. Conversely, we found a dramatically increased F/B ratio, which coincided with an increase in genera of *Lactobacillus* and a decrease in genera of *Bacteroidetes* in red-light treatment. Although Lactobacillus displayed strong probiotic characteristics, its overgrowth may occupy intestinal niches and overproduce lactic acid, limiting the growth of other commensal bacteria and impairing overall microbial diversity, which was linked to poorer growth outcomes. This comparison highlights that a balanced F/B ratio, as induced by blue light, is more reflective of a healthy, resilient, and functionally versatile gut ecosystem that favors broiler productivity than an excessively high ratio dominated by a single genus. This beneficial microbial profile appeared to be driven by the enrichment of specific melatonin-sensitive bacteria with defined probiotic functions. We identified *Ligilactobacillus aviarius* and *Akkermansia muciniphila* as melatonin-driven bacteria whose abundance fluctuated with the plasma melatonin level. These bacteria are promising probiotics, wherein *Ligilactobacillus aviarius* encodes a glycosyltransferase that converts amylose starch into isomalto-/malto-polysaccharides (IMMP), which has digestibility resistance to improve its distribution to the large intestine [52]. Therefore, IMMP serves as a prebiotic source for the bacterial community in the large intestine to produce short-chain fatty acids (SCFAs) [53]. However, high-abundant *Ligilactobacillus aviarius* significantly decreased starch content, whereas the IMMP level has no obvious rise in our study (Figure S5). In addition, *Akkermansia muciniphila* is a mucin-degrading intestinal symbiont, which is characterized as protecting the intestinal mucosal barrier in poultry. Consistent with the foregoing results, we observe an increase in the number of goblet cells, accompanied by *Akkermansia muciniphila* enrichment in the broiler’s jejunum [54] (Figure S6). This result insinuates that *Ligilactobacillus aviarius* and *Akkermansia muciniphila* may participate in broiler gut health maintenance but needs further research.

Defining and characterizing various microbiota metabolites can help to understand the interaction between host and microbiota. In our study, metabolomic profiling revealed that the blue-light-enriched probiotics, such as *Akkermansia muciniphila*, *Bifidobacterium longum*, and *Ligilactobacillus aviaries*, were associated with increased production of indole propionic acid (IPA), a microbially derived tryptophan metabolite that positively correlates with members of the *Lactobacillaceae* and *Bifidobacteriaceae* families [55,56]. This finding is functionally relevant, as IPA is inextricably linked to gut barrier function by up-regulating mucin expression and enhancing the gut mucus barrier [57]. Beyond its role in barrier integrity, IPA also influences host cholesterol metabolism by modulating cholesterol transport [58,59]. In line with this, our metabolomics data revealed a corresponding decline in jejunal cholesterol levels in IPA-enriched chickens. Cholesterol is synthesized from its precursor 7-dehydrocholesterol, metabolized to 7α-hydroxycholesterol, and converted to cholic acid and other primary bile acids. The resulting primary bile acids are further converted by gut microbes into secondary bile acids, notably, 7-ketodeoxycholic acid and 12-ketodeoxycholic acid, which play critical roles in cholesterol and lipid metabolism and in host–microbe crosstalk [60,61]. Thus, the IPA-associated shift in cholesterol metabolism likely promotes secondary bile acid production, which in turn enhances lipid digestion and energy harvest, which could be a key contributor to improved growth performance. This interpretation is further supported by evidence that *Akkermansia muciniphila* modulates host cholesterol metabolism through its metabolites or via direct crosstalk with host cells [62,63,64]. Taken together, the melatonin-induced reduction in jejunal oxidative stress selectively enriches *Akkermansia muciniphila*, *Bifidobacterium longum*, and *Ligilactobacillus aviarius* through the mechanisms discussed above. Importantly, this compositional shift has profound functional consequences, as revealed by our metagenomic and metabolomic analyses. Specifically, the enrichment of these probiotics is associated with increased production of IPA, which could enhance nutrient absorption, reduce inflammation, and improve energy harvest, which directly translates into the significant weight gain and production efficiency observed in blue-light-treated broilers.

It is documented that monochromatic light regulates the assembly, stability and evolution of microbial communities in avian species by various pathways [19,20]. Apart from those melatonin-driven gut bacteria enriched in the blue-light group, we also identify *Liquorilactobacillus* and *Lactiplantibacillus* as blue-light-sensitive bacteria. These bacteria retain high abundance in pinealectomized chicken. In fact, photoreceptor cells reside in the avian retina and hypothalamus, as well, which triggers synthesis of steroid hormones with light stimulation. In this sense, chicken can detect blue light by the retina and hypothalamus rather than the pineal gland to directly or indirectly manipulate gut microbiota [65]. Specifically, photostimulation detected by the photosensitive retinal ganglion cells (ipRGCs) or deep-brain photoreceptors in the hypothalamus is transmitted to the suprachiasmatic nucleus (SCN), thereby activating the central circadian oscillator and rhythmic pineal melatonin secretion [11]. Systemic melatonin could subsequently reshape the gut microbiome. Beside driving melatonin synthesis, light can influence the gut microbiota composition through complex, multi-stage neural computations within these extra-pineal photoreceptive organs. In brief, the photosensitive retinal ganglion cells (ipRGCs) could release both excitatory glutamate and inhibitory GABA neurotransmitters, which connect broadly within SCN. These inputs to the SCN may participate in feeding-related entrainment pathways that ultimately shape the gut microbial composition [66,67]. Notably, the avian hypothalamus has been verified to directly respond to light exposure, regulating physiological processes such as feeding, metabolism and reproduction. These photoreceptive cells likely modulate the hypothalamic–adrenal/thyroid axes through local neural circuits, thereby influencing intestinal microenvironment and microbiota composition via hormonal or neurochemical pathways [68,69]. However, the light–dark circle (23L:1D) in our study has been verified to disrupted the circadian rhythm. In this sense, we inferred that *Liquorilactobacillus* and *Lactiplantibacillus* may be driven by pathways directly activated by blue-light-sensitive photoreceptors distributed in the retina and hypothalamus of broilers [70]. Moreover, we find a selective sensitivity of the chicken gut microbiota to middle-(green) or long-wavelength (red) light. In our research, green light increases *Blautia* and *Bacillus*, which are an essential group related to producing a wide range of secondary metabolites known for their beneficial effects on broiler growth. The abundance of *Lactobacillus*, *Lacticaseibacillus*, *Staphylococcus*, and *Clostridioides* increase significantly with light wavelength, wherein *Staphylococcus* is a pathogen, and *Lactobacillus* can influence the adaptive immune responses and improve production parameters. These findings suggested that specific light wavelength modulation could indubitably serve as a novel strategy of replacing antibiotics in commercial poultry production.

In fact, broilers are typically reared under white light, which comprises a combination of multiple wavelengths. Specific light wavelength could play a dominant role in particular physiological pathways. Therefore, farmers can select different monochromatic light or adjust the proportion of monochromatic light in white light for distinct breeding needs. Our results demonstrated that monochromatic blue light was sufficient and highly effective in altering gut microbiota composition by manipulating pineal-derived melatonin production. We chose to use monochromatic light in this study to avoid potential interference that might occur with polychromatic light, thereby allowing clearer delineation of our core mechanism. However, previous studies have reported that combined light regimes, such as blue–green light, also confer benefits to broiler health [71]. This raises two important forward-looking questions: (1) In combined light treatment, does the specific blue light elucidated in our study remain the dominant effect? (2) Could a blue–green-light combination provide a synergistic effect on inducing melatonin production or optimizing microbial community composition? Based on our preliminary result, future research involving systematic comparisons of combined lights and monochromatic light would enable the design of more precise and efficient lighting strategies for broiler breeding.

Additionally, several limitations should be acknowledged in our study. First of all, our investigation focused exclusively on the jejunum of 35-day-old broilers, as this segment was central to our oxidative stress and microbiota analyses. However, melatonin receptors are distributed throughout the chicken intestine, and other gut segments may also respond to light-induced melatonin signaling. We also acknowledge that our pharmacological inhibition experiments using Luzindole and ML385 suggest the involvement of Mel1a and Nrf2 signaling, but do not definitively establish receptor specificity due to potential off-target effects. However, these were not examined in the current study. Further genetic approaches would be required to confirm the specific role of this receptor in different intestinal segments. Second, we did not evaluate the potential effects of long-term blue-light exposure on broiler behavior, stress or ocular health. Broiler behavior and stress were influenced by light color. For example, broilers reared under blue light were calmer with lower stress and fear; thereby, the welfare of poultry and subsequent body weight gain could be improved [72,73]. On the other hand, blue light is harmful to eyes. It is known that prolonged blue-light exposure could provoke photochemical reactions and cause temporary or permanent damage to the retina in mammals and humans [74]. Although the avian visual system differs from mammals, this remains a crucial consideration for long-term broiler welfare [75]. Third, our data correlating plasma melatonin with gut microbial composition do not establish causation. While we observed significant associations between melatonin levels and specific bacterial taxa, we did not perform exogenous melatonin administration to confirm that melatonin directly drives these microbial changes. Future studies employing exogenous melatonin supplementation in sham-operated birds or melatonin receptor antagonist treatment in pinealectomized birds are warranted to establish a causal relationship between melatonin and gut microbiota modulation. Most importantly, the effect of three photoreceptive organs on modulating gut microbiome composition were not mutually exclusive but likely interact and converge, as we discussed previous. Therefore, future studies should incorporate more longitudinal and multidimensional assessments, such as: (1) analyzing the microbial profile of other intestine segments; (2) quantifying animal behavior and welfare indicators under different light colors using infrared cameras and a scan sampling technique; (3) systematically evaluating the impact of monochromatic light on retinal structure and visual function through histopathological and functional examinations; (4) investigating the regulatory role of extra-pineal photoreceptive organs in reshaping gut microbiota in commercial broilers and elucidating the potential crosstalk among these photoreceptive organs.

## 5. Conclusions

**Figure 7:**
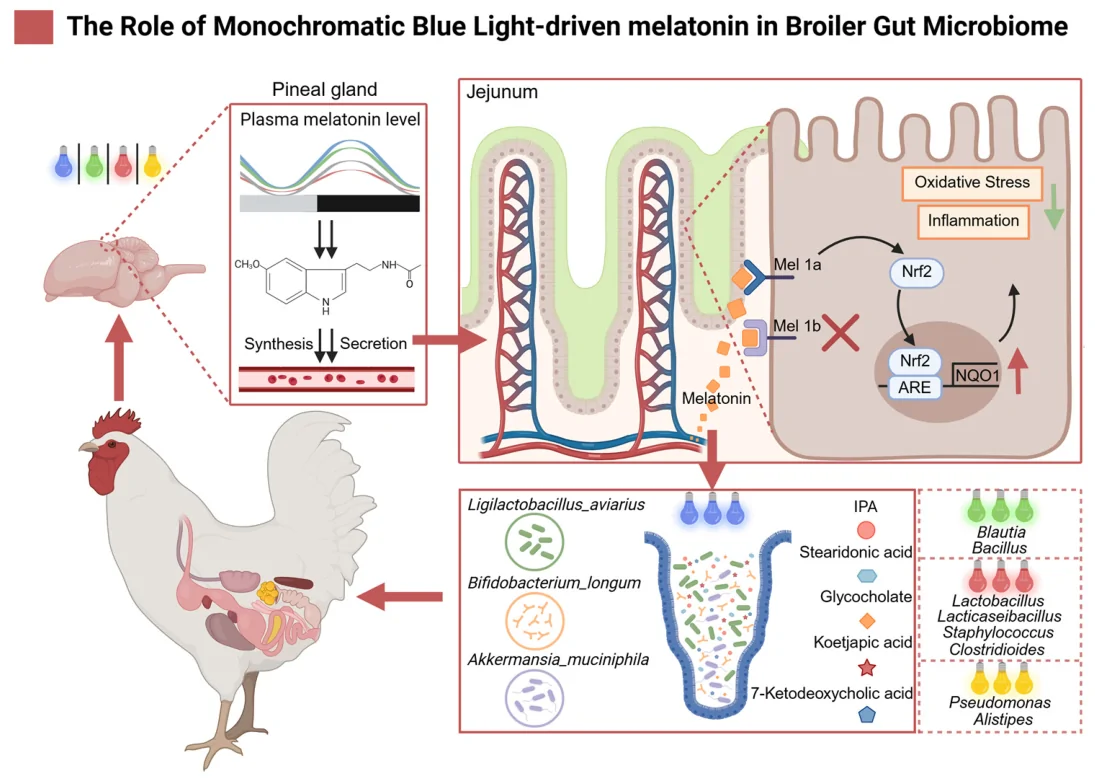
**Schematic diagram of the protective role of monochromatic blue-light-driven melatonin on jejunal microbiome and their metabolites.** Briefly, monochromatic blue light increased circulating melatonin level and then activated Nrf2-NQO1 pathway via melatonin receptor 1a in broiler jejunum to reduce oxidative stress. Lower oxidative stress altered gut microbiome composition and its metabolites to improve jejunal function and growth performance. In detail, melatonin upregulated the population of *Akkermansia muciniphila*, *Bifidobacterium longum*, *Ligilactobacillus aviarius* and the production of the Indole propionic acid, Stearidonic acid, Glycocholate, Koetjapic acid and 7-Ketodeoxycholic acid. We also found that monochromatic green light increased the relative abundance of *Blautia* and *Bacillus*, while *Lactobacillus*, *Lacticaseibacillus*, *Staphylococcus* and *Clostridioides* were more prevalent in monochromatic red light. Moreover, *Pseudomonas* and *Alistipes* were more abundant in white light.

Taken together, our finding indicates that the associations between the broiler jejunal microbiome composition and monochromatic light are robust, with distinct species dominating under different light colors. Notably, using the pinealectomy model to establish melatonin dependency, we provide evidence that the key bacteria *Akkermansia muciniphila*, *Bifidobacterium longum* and *Ligilactobacillus aviarius* prevailing in blue light are causally linked to elevated plasma melatonin. Mechanistically, monochromatic blue light significantly increases systemic melatonin, which may bind to its membrane receptor Mel1a and activate the downstream Nrf2/NQO1 signaling pathway to reduce oxidative stress in the broiler jejunum, potentially favoring the colonization of these melatonin-sensitive probiotics. In turn, these specific bacteria are associated with numerous beneficial metabolites, including those that modulate body weight and promote broiler growth (Figure 7). Collectively, our findings not only advance the fundamental understanding of how light signals are transduced through melatonin to reshape the gut microbiome, but also offer a putative mechanistic framework for optimizing light management strategies in commercial broiler production.

## Figures and Tables

**Table 1:** Primer sequences.

Genes	Primer Sequence (5′-3′)	Accession No.
*AANAT*	F: GGACCAGGACAGGCTCAG R: CGAAACCACACTTCTCGTAG	NM_204305.2
*ASMT*	F: ATTCAATCCCTGAAGCTGACCTC R: TCTTCACTCAGGAGCGATTCAAC	NM_205343.2
*IL-10*	F: AGATGCTGCGCTTCTACACA R: CCCATGCTCTGCTGATGACT	NM_001004414.4
*IL-6*	F: CTCCTCGCCAATCTGAAGTC R: AGGCACTGAAACTCCTGGTCT	NM_204628.2
*IL-1β*	F: TGCCTGCAGAAGAAGCCTCG R: CTCCGCAGCAGTTTGGTCAT	NM_204524.2
*IFN-γ*	F: GGATGCCACCTTCTCTCACG R: TGGTGTCGTTGAAGGAGCAA	XM_046936227.1
*Nrf2*	F: CTGCTAGTGGATGGCGAGAC R: CTCCGAGTTCTCCCCGAAAG	XM_046943483.1
*HO-1*	F: CAACGCTGAAAGCATGTCCC R: GACCAGCTTGAACTCGTGGA	NM_205344.2
*NQO1*	F: ATAGGGTTCAATGCCGTGCT R: TCGTAGACAAAGCACTCGGG	NM_001277619.2
*Keap1*	F: ATGTACCAGATCGACAGCGT R: AACTCCTCCTGCTTGGAGAC	XM_040683960.2
*β-TrCP*	F: TGCTCCACCCAACTCATTCTACAG R: CACACCGCCAGTTTACTCTATTG	XM_040675169.2
*GAPDH*	F: ATCACAGCCACACAGAAGACG R: TGACTTTCCCCACAGCCTTA	NM_204305.2

F = Forward primer; R = Reverse primer.

## Data Availability

The raw sequencing data generated in this study have been deposited in the Genome Sequence Archive (https://ngdc.cncb.ac.cn/gsa/s/9d4Ghn14, accessed on 11 October 2025) in the National Genomics Data Center with the accession number CRA031141, and the metabolome data are deposited in OMIX with the accession number OMIX012207 (https://ngdc.cncb.ac.cn/omix/preview/ruDBABTamM, accessed on 9 October 2025).

## Supplementary Materials

The following supporting information can be downloaded at https://www.mdpi.com/article/10.3390/antiox15091204/s1, Figure S1: Relative abundance of monochromatic light-sensitive microbiome; Figure S2: Plasma melatonin increased gut microbiome health index (GMHI) of broiler jejunum; Figure S3: Metagenome-enriched KEGG metabolic pathway-related functional genes; Figure S4: Tryptophan metabolism in broiler jejunum; Figure S5: In vitro experiment on CSIEC; Figure S6: Ligilactobaciilus.aviarius participated in generating Isomaltose; Figure S7: Increased goblet cells accompanied by Akkermansia_muciniphila enrichment in the broiler’s jejunum.
