# Supplementary material for: Monochromatic Blue Light Enhances Antioxidant Status and Remodels the Gut Microbiome in Association with Increased Plasma Melatonin in Broiler Chickens
Source: Antioxidants (Basel). 2026 Sep 20;15(9):1204. doi: 10.3390/antiox15091204 (PMC13603340; doi:10.3390/antiox15091204)
Supplement: Supplementary file 1 [file antioxidants-15-01204-s001.zip › antioxidants-4521953-supplementary.pdf]

Supplementary Materials

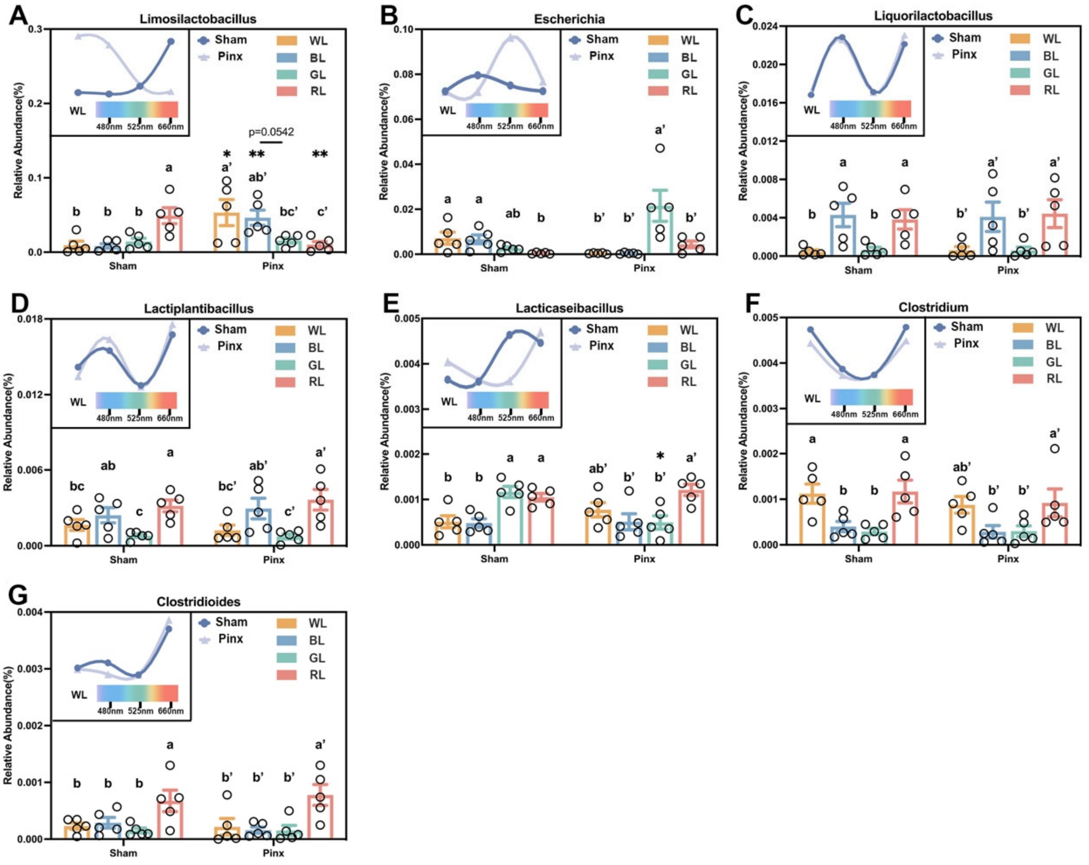

Figure. S1. Relative abundance of monochromatic light-sensitive microbiome. (A-G) Relative abundance of key bacteria (*Limosilactobacillus, Escherichia, Liquorilactobacillus, Lactiplantibacillus, Lacticaseibacillus, Clostridium, Clostridioides*) between sham-operation and pinealectomy group under monochromatic lights.

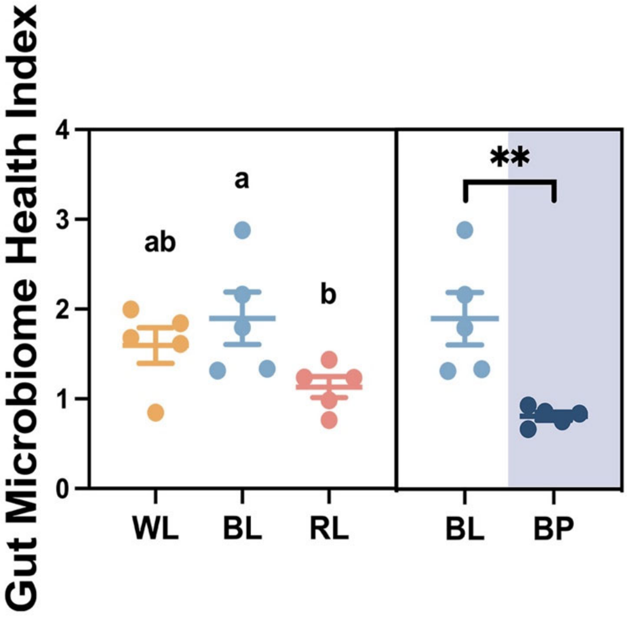

Figure. S2. Plasma melatonin increased gut microbiome health index (GMHI) of broiler jejunum.

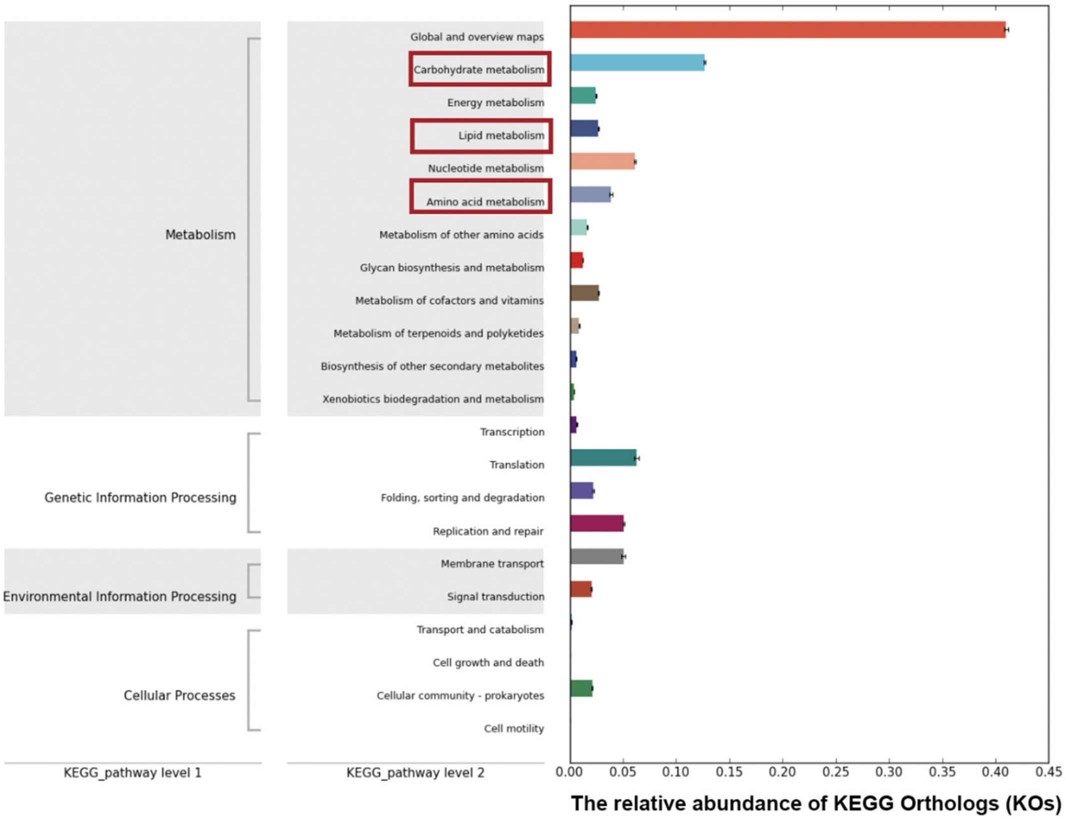

Figure. S3. Metagenome-enriched KEGG metabolic pathway-related functional genes.

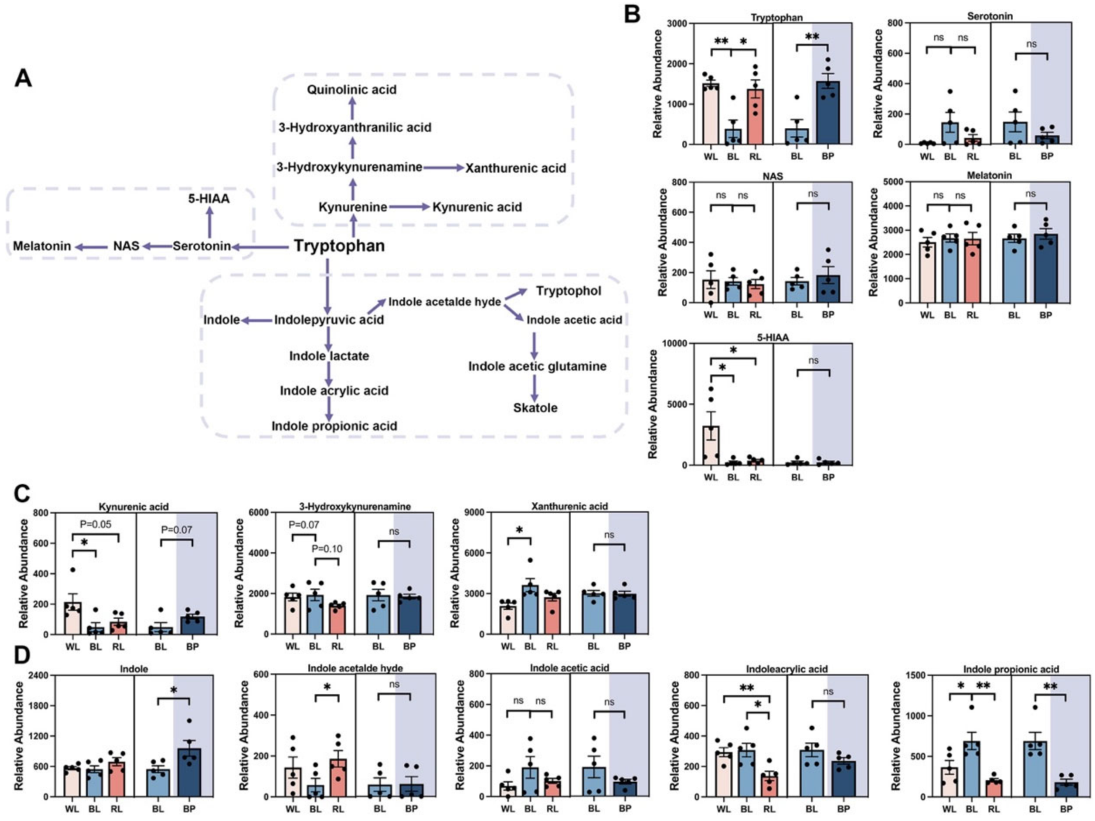

Figure. S4. Tryptophan metabolism in broiler jejunum. (A) Schematic of the three major Tryptophan metabolism pathways. (B) Melatonin pathways and its relevant metabolites. (C) Kynurenic acid pathways and its relevant metabolites. (D) Indole pathways and its relevant metabolites.

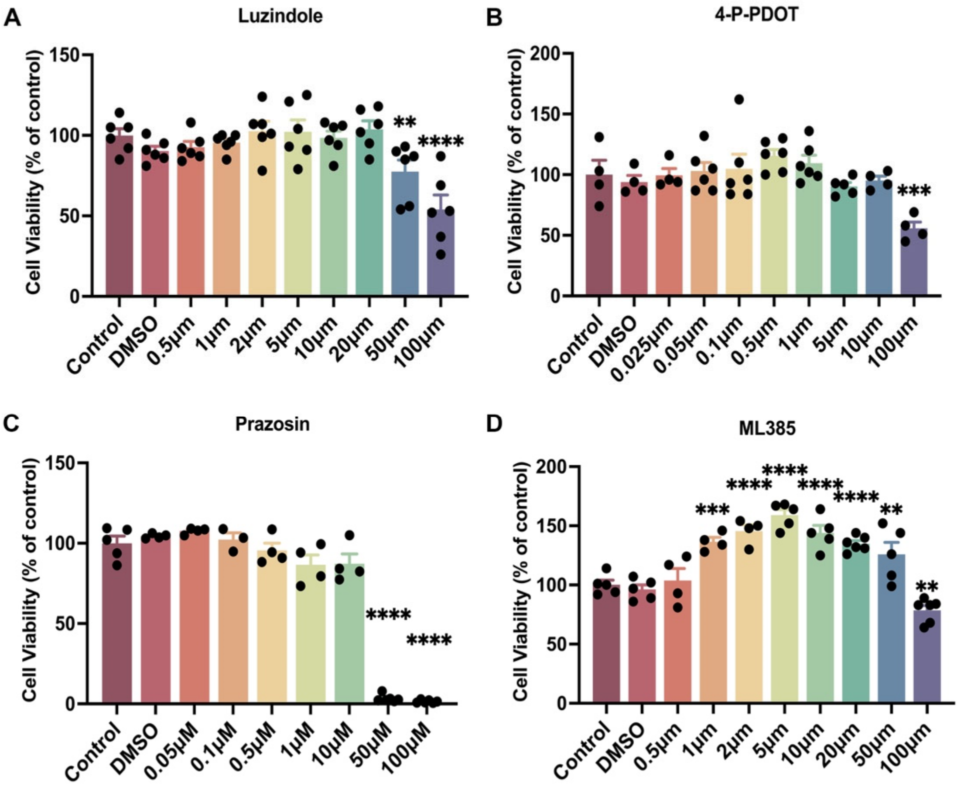

Figure. S5. In vitro experiment on CSIEC. (A-D) Wide range of safe concentration (Luzindole, 4P-P-DOT, Prazosin and ML385).

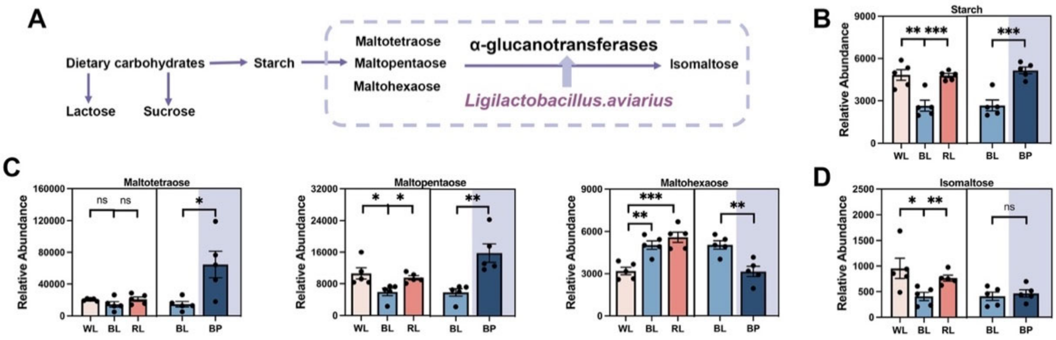

Figure. S6. *Ligilactobaciilus.aviarius* participated in generating Isomaltose. (A) Summary of the metabolic pathway from Starch to Isomaltose. (B) Relative abundance of Starch. (C) Relative abundance of maltotetraose, maltopentaose and maltohexaose. (D) Relative abundance of isomaltose.

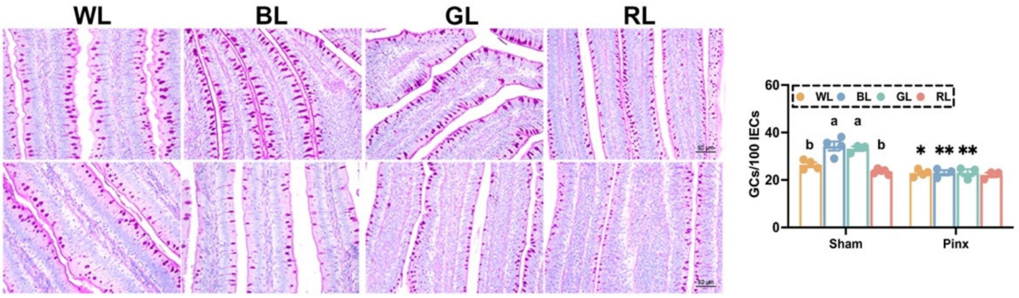

Figure. S7. Increased goblet cells accompanied by *Akkermansia_muciniphila* enrichment in the broiler’s jejunum.
